# Non-Fragile Observer-Based Adaptive Integral Sliding Mode Control for a Class of T-S Fuzzy Descriptor Systems With Unmeasurable Premise Variables

**DOI:** 10.3389/fnbot.2022.820389

**Published:** 2022-07-22

**Authors:** Xiaorong Huang, Anca L. Ralescu, Yiqiang Peng, Hongli Gao, Shulei Sun

**Affiliations:** ^1^Vehicle Measurement, Control and Safety Key Laboratory of Sichuan Province, School of Automobile and Transportation, Xihua University, Chengdu, China; ^2^Department of Electrical Engineering and Computer Science, University of Cincinnati, Cincinnati, OH, United States; ^3^School of Mechanical Engineering, Southwest Jiaotong University, Chengdu, China

**Keywords:** T-S fuzzy descriptor systems, non-fragile control, observer design, integral sliding mode control, nonparallel distributed compensation control, unmeasurable premise variables

## Abstract

The issue of non-fragile observer-based adaptive integral sliding mode control for a class of Takagi–Sugeno (T-S) fuzzy descriptor systems with uncertainties and unmeasurable premise variables is investigated. General nonlinear systems are represented by nonlinear T-S fuzzy descriptor models, because premise variables depend on unmeasurable system states and fuzzy models have different derivative matrices. By introducing the system state derivative as an auxiliary state vector, original fuzzy descriptor systems are transformed into augmented systems for which system properties remain the same. First, a novel integral sliding surface, which includes estimated states of the sliding mode observer and controller gain matrices, is designed to obtain estimation error dynamics and sliding mode dynamics. Then, some sufficient linear matrix inequality (LMI) conditions for designing the observer and the controller gains are derived using the appropriate fuzzy Lyapunov functions and Lyapunov theory. This approach yields asymptotically stable sliding mode dynamics. Corresponding auxiliary variables are introduced, and the Finsler's lemma is employed to eliminate coupling of controller gain matrices, observer gain matrices, Lyapunov function matrices, and/or observer gain perturbations. An observer-based integral sliding mode control strategy is obtained to assure that reachability conditions are satisfied. Moreover, a non-fragile observer and a non-fragile adaptive controller are developed to compensate for system uncertainties and perturbations in both the observer and the controller. Finally, example results are presented to illustrate the effectiveness and merits of the proposed method.

## Introduction

In recent decades, control synthesis, stability analysis, and observer design for nonlinear systems have received important consideration because of their wide application in practice, and demands for reliability and performance have been increasingly enhanced. Nevertheless, it has become challenging to systematically design and analyze such systems. The Takagi–Sugeno (T-S) fuzzy system, also known as the type III fuzzy model, proposed by Japanese scholars in 1985 (Takagi and Sugeno, 1985) provides a general approach to approximate any smooth nonlinear system with an arbitrary degree of accuracy but without complex mathematical equations. Through the use of the T-S fuzzy model approach, systematic analysis and synthesis of nonlinear systems can be performed based on classical control theory (Lv et al., 2019), modern control theory (Zhang Z. et al., 2019), and intelligent control theory (Sun et al., 2007; Cervantes et al., 2016). Due to their strong approximation capabilities and good tolerance to uncertainty and imprecision, T-S fuzzy control techniques have been widely used in the area of intelligent control of robotics, i.e., for robot manipulators (Fan et al., 2020), nonlinear flexible link robots (Shams and Seyedtabaii, 2020), and planar parallel robots (Vermeiren et al., 2012) among others. Therefore, the T-S fuzzy model is an effective intelligent method for modeling and analyzing robotic systems.

In practical systems, all state variables cannot always be measured by sensors, and in other cases, the sensors used to measure system states are expensive; however, the state variables are indispensable to the design of system controllers and analyses of system stability. Thus, observer design plays an important role in stability analysis and control synthesis for nonlinear systems. A fuzzy observer was first proposed by Tanaka and Sano in 1994 (Tanaka and Sano, 1994), and observers have since received extensive research attention. Various problems involving fuzzy observers have been studied in the literature. The researchers in (Tong and Li, 2002; Asemani and Majd, 2013) studied an observer-based controller design, and the observer and controller were built simultaneously. The sliding mode fuzzy observers in (Shen et al., 2011) were designed to address the problem of fault-tolerant control for T-S fuzzy systems with actuator faults. Based on the Lyapunov method, sufficient conditions for an unknown input T-S observer (Chadli and Karimi, 2012) were given in a linear matrix inequality (LMI) formulation. The novel fuzzy learning observer in (You et al., 2019) was constructed to achieve simultaneous reconstruction of system states and actuator faults for T-S fuzzy systems with time-varying delays. However, the above methods for T-S fuzzy systems are difficult to implement in practice because of their high complexity. Compared with T-S fuzzy systems, T-S fuzzy descriptor systems have the following advantages: they effectively reduce the number of fuzzy rules in a nonlinear system, and they can describe a wider variety of nonlinear systems. Hence, the observer design problem for the traditional nonlinear T-S descriptor system is considered in this study.

In Li and Zhang (2018); Zhang et al. (2018); Zhang J. et al. (2019), the authors designed a reduced-order robust observer, a robust adaptive sliding mode observer, and a robust H∞ sliding mode observer; additionally, an observer-based sliding mode controller was proposed for T-S fuzzy descriptor systems with time-varying delay. Observer-based integral sliding mode control strategies were developed in (Li et al., 2018). An adaptive fuzzy observer in (Kharrat et al., 2018), a novel fuzzy descriptor learning observer in (Jia et al., 2015), a robust fuzzy descriptor observer in (Brahim et al., 2017), and some T-S descriptor observers in (López-Estrada et al., 2017; Haj Brahim et al., 2019) were constructed to achieve simultaneous reconstruction of system states and actuator/sensor faults. A fault-tolerant control scheme was derived based on Lyapunov asymptotic stability. Robust observer-based output feedback control and the robustness issue were addressed in (Liu et al., 2013) to avoid control performance deterioration or instability due to disturbances or approximation errors in the system. The design process of a T-S fuzzy observer was extended to a class of T-S descriptor systems with unmeasurable premise variables in (Soulami et al., 2015). When designing a fuzzy observer, it is important to have a clear understanding of the relationship between premise variables and estimated system states. From the literature (Liu et al., 2013; Jia et al., 2015; Soulami et al., 2015; Brahim et al., 2017; López-Estrada et al., 2017; Kharrat et al., 2018; Li and Zhang, 2018; Li et al., 2018; Zhang et al., 2018; Haj Brahim et al., 2019; Zhang J. et al., 2019), we can conclude that two cases exist to describe this relationship. Case A: premise variables are not dependent on system states estimated by the fuzzy observer, and case B: premise variables depend on system states estimated by the fuzzy observer. The design process of the controller and the observer is relatively simple in case A compared to the approach in case B; therefore, more studies have focused on case A such as (Liu et al., 2013; Jia et al., 2015; Kharrat et al., 2018; Li and Zhang, 2018; Li et al., 2018; Zhang et al., 2018; Zhang J. et al., 2019). However, it should be noted that premise variables usually depend on unmeasurable system states in practical systems. Therefore, case A has more restrictive conditions that limit its application to various systems. Some researchers in (Soulami et al., 2015; Brahim et al., 2017; López-Estrada et al., 2017; Haj Brahim et al., 2019) studied the observer design for T-S fuzzy descriptor systems with unmeasurable premise variables.

Sliding mode control (SMC), as a type of variable structure control method, can effectively control systems with nonlinearities and uncertainties because of its beneficial characteristics such as fast response, good transience, and strong robustness. In recent decades, many significant results (Vu et al., 2012; Van et al., 2013; Li and Zhang, 2018; Zhang et al., 2018; Wu et al., 2019; Zhang J. et al., 2019) based on fuzzy systems have been reported in studies on sliding mode observer design and observer-based SMC. A novel fuzzy second-order sliding mode observer was designed to estimate robot velocity, and a new fuzzy second-order sliding mode strategy based on T-S fuzzy models was proposed to track the expected motion in (Van et al., 2013). A T-S fuzzy-model-based sliding mode controller was developed for surface-mounted permanent-magnet synchronous motors in Vu et al. (2012) considering motor parameter uncertainties and unknown external noise. In (Wu et al., 2019), the disturbance in T-S fuzzy discrete time systems was monitored by a disturbance observer. The core objective of a sliding mode observer is to design an SMC strategy for an observer system or a dynamic estimation error system. Hence, a sliding mode observer designed with the SMC method has high robustness to nonlinearities and uncertainties. From the above literature, it is clear that the traditional SMC theory requires a reaching phase to drive state trajectories to the desired sliding surface. The integral sliding mode technique can eliminate this process by implementing sliding mode motion from the initial time of the control action. Consequently, the matched uncertainties can be compensated for throughout the integral sliding mode control (ISMC) process. Therefore, the issue of applying an integral sliding mode technique to design observers and controllers for fuzzy systems has received significant attention, such as in (Jiang et al., 2018; Li et al., 2018; Kuppusamy and Joo, 2019). In (Jiang et al., 2018), a novel integral sliding surface function was proposed for the observer space of T-S fuzzy systems with semi-Markov switching and immeasurable premise variables. An integral-type fuzzy switching surface function was defined that simultaneously involved a state-dependent input matrix and a memory parameter in Kuppusamy and Joo (2019).

The above methods of controller and/or observer design for T-S descriptor systems are based on an implicit assumption that a desired controller and/or observer can be realized exactly. However, in practical applications, it is impossible to implement an ideal designed controller or observer because of round-off errors in numerical computations, digital-to-analog conversion errors, the finite word length used in digital computer systems, and other factors. Therefore, a significant issue is determining how to design a controller and an observer that are able to tolerate some uncertainties in various processes, and is called non-fragile control. The problem of non-fragile controller design has been addressed, and a non-fragile guaranteed cost controller (Chen and Li, 2013), a non-fragile fuzzy dissipative static output feedback control (Guan and Liu, 2016), and a non-fragile robust H∞ control (Zhang et al., 2007) have been investigated. The researchers in (Li X. et al., 2017; Duan et al., 2019) focused on the issue of non-fragile observer design.

Although a considerable effort has been devoted to fuzzy observer analysis, ISMC design, and non-fragile control for fuzzy systems and some effective solutions have been developed, there are still some limitations in the existing research. First, the above studies (Jia et al., 2015; Soulami et al., 2015; Brahim et al., 2017; López-Estrada et al., 2017; Kharrat et al., 2018; Li and Zhang, 2018; Zhang et al., 2018; Haj Brahim et al., 2019; Zhang J. et al., 2019) mainly discussed T-S fuzzy descriptor systems with the same derivative matrix *E*. Other studies (Taniguchi et al., 2000; Chen et al., 2009) showed that T-S fuzzy descriptor systems with different derivative matrices are more useful for modeling and analyzing the complexity of nonlinear systems than T-S fuzzy descriptor systems with the same derivative matrices. To the best of the authors' knowledge, to date, the problem of observer design for T-S fuzzy descriptor systems with unmeasurable premise variables and a different derivative matrix *E* has not been previously studied. Second, from what we can ascertain, the existing integral sliding mode observer and controller in (Jiang et al., 2018; Li et al., 2018; Kuppusamy and Joo, 2019) were designed only for T-S fuzzy systems or T-S descriptor systems with measurable premise variables. The problem of observer-based adaptive ISMC for T-S fuzzy descriptor systems with unmeasurable premise variables and uncertainties has not been previously studied. Finally, fruitful results have been obtained for non-fragile controllers and/or observers for T-S fuzzy systems such as those in (Zhang et al., 2007; Chen and Li, 2013; Guan and Liu, 2016; Li X. et al., 2017; Duan et al., 2019), but these results generally lack corresponding techniques for T-S fuzzy descriptor systems.

Motivated by the abovementioned discussion, in this article, we study non-fragile observer-based ISMC problems for T-S fuzzy descriptor systems with unmeasurable premise variables and uncertainties. The main contributions of the proposed control method are as follows.

1) In accordance with the nonparallel distributed compensation control (non-PDC) method and the ISMC theory, an observer-based integral sliding mode controller is developed for T-S fuzzy descriptor systems with unmeasurable premise variables.
2) A non-fragile integral sliding mode observer and a non-fragile observer-based sliding mode controller are constructed. In addition, system uncertainties and perturbations in both the observer structure and the controller structure are compensated for by an adaptive controller.
3) Auxiliary variables are introduced into the system with an augmented method to eliminate the coupling of Lyapunov function matrices, observer gain matrices, and/or observer gain perturbations. Moreover, a fuzzy Lyapunov function containing information for system state estimation and system state estimation error is designed to guarantee the asymptotic stability of the closed-loop system. The auxiliary variables and the fuzzy Lyapunov function produce unconservative results.

This article is organized as follows. Section Problem Formulation and Preliminary Analysis describes the system, clarifies the problem formulation, and gives relevant preliminary information. Then, in Section Non-Fragile Observer-Based ISMC for T-S Fuzzy Descriptor Systems, a non-fragile observer and a non-fragile controller are constructed. A simulation example is presented to validate the accuracy and effectiveness of the proposed method in Section Examples. Finally, conclusions are drawn in Section Conclusions.

Notation: in this study, *R*^*m*^ and *R*^*n*×*m*^ denote the n-dimensional real Euclidean space and the set of *n*×*m* matrices with real elements, respectively. *I* is the identity matrix with appropriate dimensions. For clarity, the following definitions are given: $\gamma_{h}=\sum_{i=1}^{r}h_{i}\left(▪\right)\gamma_{i}$, $\gamma_{h}^{-1}={\left(\sum_{i=1}^{r}h_{i}\left(▪\right)\gamma_{i}\right)}^{-1}$, $\gamma_{h,h}=\sum_{i=1}^{r}\sum_{j=1}^{r}h_{i}\left(▪\right)h_{j}\left(▪\right)\gamma_{i,j}$, $\gamma_{\overset{⌢}{h}}=\sum_{i=1}^{r}h_{i}\left(\overset{⌢}{\xi }\left(t\right)\right)\gamma_{i}$, *A* + * = *A* + *A*^*T*^, He(*A*)=*A* + *A*^*T*^, and $\left[\begin{array}{cc} A & *\\ B & C \end{array}\right]=\left[\begin{array}{cc} A & B^{T}\\ B & C \end{array}\right]$.

## Problem Formulation And Preliminary Analysis

In this study, T-S fuzzy descriptor systems are used to approximate various complex nonlinear robotics, i.e., robot manipulators (Fan et al., 2020), planar parallel robots (Vermeiren et al., 2012), an overhead crane system (Chen et al., 2009), a ball and beam system (Li H. et al., 2017), and a nonlinear active vehicle suspension system (Li et al., 2012). Therefore, consider a class of uncertain robotic systems that can be represented by the following T-S fuzzy descriptor systems with uncertainties:


(1) $$\begin{array}{l} \;\sum_{k=1}^{r_{e}}v_{k}\left(\xi \left(t\right)\right)\left(E_{k}+\text{Δ}E\right)\dot{x}\left(t\right)\\ =\sum_{i=1}^{r}h_{i}\left(z\left(t\right)\right)\left\{\left(A_{i}+\text{Δ}A\right)x\left(t\right)+Bu\left(t\right)\right\}, \end{array}$$

$$\begin{array}{l} y\left(t\right)=\sum_{i=1}^{r}h_{i}\left(z\left(t\right)\right)\left(C_{i}x\left(t\right)\right), \end{array}$$

where $x\left(t\right)=\left[\begin{array}{ccc} x_{1}\left(t\right) & \cdots & x_{n}\left(t\right) \end{array}\right]\in R^{n}$is the system state vector, *u*(*t*) ∈ *R*^*m*^ is the control input, *y*(*t*) ∈ *R*^*p*^is the system output vector, and $\xi \left(t\right)=\left[\begin{array}{ccc} \xi_{1}\left(t\right) & \cdots & \xi_{l}\left(t\right) \end{array}\right]\in R^{l}$ and $z\left(t\right)=\left[\begin{array}{ccc} z_{1}\left(t\right) & \cdots & z_{q}\left(t\right) \end{array}\right]\in R^{q}$are the unmeasurable premise vectors. *v*_*k*_(ξ(*t*)), *k* = 1, 2, ⋯ , *r*_*e*_ and *h*_*i*_(*z*(*t*)), *i* = 1, 2, ⋯ , *r* are fuzzy membership functions on the left and right-hand sides, respectively. $A_{i}\in R^{n\times n}$, $E_{k}\in R^{n\times n}$, *B* ∈ *R*^*n*×*m*^, and $C_{i}\in R^{p\times n}$ are the system matrices. Δ*E* ∈ *R*^*n*×*n*^ and Δ*A* ∈ *R*^*n*×*n*^ are the system uncertainties. In many practical cases, there are two main sources of system uncertainties. On the one hand, mechanical devices may carry various tools or goods for various operations; thus, the system's mass, center of mass, and other coefficients tend to change with load. On the other hand, dynamic model and system parameters are challenging to accurately obtain either through theoretical methods or by experimental measurements. In many practical cases, *h*_*i*_(*z*(*t*)) and *v*_*k*_(ξ(*t*)) are different, i.e., an inverted pendulum on a cart (Li et al., 2018), an overhead crane system (Li H. et al., 2017), a ball and beam system (Li X. et al., 2017), or a nonlinear active vehicle suspension system (Li et al., 2012).

Without loss of generality, some assumptions are introduced as follows:

Assumption 1. $\sum_{k=1}^{r_{e}}v_{k}\left(\xi \left(t\right)\right)\left(E_{k}+\text{Δ}E\right)$is nonsingular.

Assumption 2. Δ*E*andΔ*A*are uncertainties satisfying Δ*E* = *M*_*E*_*F*_*E*_(*t*)*N*_*E*_ andΔ*A* = *M*_*A*_*F*_*A*_(*t*)*N*_*A*_, where *M*_*E*_, *N*_*E*_, *M*_*A*_, and *N*_*A*_are known real constant matrices and *F*_*E*_(*t*)and*F*_*A*_(*t*) are unknown time-varying matrices that satisfy $F_{E}^{T}\left(t\right)F_{E}\left(t\right)\leq I$ and $F_{A}^{T}\left(t\right)F_{A}\left(t\right)\leq I$, respectively.

By defining $X^{*}\left(t\right)={\left[\begin{array}{cc} x^{T}\left(t\right) & {\dot{x}}^{T}\left(t\right) \end{array}\right]}^{T}$, the T-S fuzzy descriptor system (1) can be transformed into an augmented form as follows:


(2) $$\begin{array}{l} E^{*}{\dot{X}}^{*}\left(t\right)=\sum_{k=1}^{r_{e}}\sum_{i=1}^{r}v_{k}\left(\xi \left(t\right)\right)h_{i}\left(z\left(t\right)\right)\left\{\left(A_{ki}^{*}+\text{Δ}A^{*}\right)X^{*}\left(t\right)+B^{*}u\left(t\right)\right\}, \end{array}$$

$$\begin{array}{l} y\left(t\right)=\sum_{i=1}^{r}h_{i}\left(z\left(t\right)\right)C_{i}^{*}X^{*}\left(t\right), \end{array}$$

where $E^{*}=\left[\begin{array}{cc} I & 0\\ 0 & 0 \end{array}\right]$,$A_{ki}^{*}=\left[\begin{array}{cc} 0 & I\\ A_{i} & -E_{k} \end{array}\right]$,$M=\left[\begin{array}{cc} 0 & 0\\ M_{A} & -M_{E} \end{array}\right]$,$\text{Δ}A^{*}=\left[\begin{array}{cc} 0 & I\\ \text{Δ}A & -\text{Δ}E \end{array}\right]=MF\left(t\right)N$, $F\left(t\right)=\left[\begin{array}{cc} F_{A}\left(t\right) & 0\\ 0 & F_{E}\left(t\right) \end{array}\right]$, $N=\left[\begin{array}{cc} N_{A} & 0\\ 0 & N_{E} \end{array}\right]$, $B^{\text{*}}=\left[\begin{array}{c} 0\\ B \end{array}\right]$, and $C_{i}^{*}=\left[\begin{array}{cc} C_{i} & 0 \end{array}\right]$.

Assumption 3. The output matrices *C*_*i*_ of the *i*th rule of the T-S fuzzy descriptor system are full row rank for all *i* = 1, ⋯ , *r*, and therefore nonsingular matrices *T*_*i*_ exist such that


$$\begin{array}{l} C_{i}T_{i}=\left[\begin{array}{cc} I & 0 \end{array}\right]. \end{array}$$

Remark 1. For any given *C*_*i*_, the corresponding *T*_*i*_ is not unique in general. One solution for *T*_*i*_, as discussed in Du and Yang (2009), is:


$$\begin{array}{l} T_{i}=\left[C_{i}^{T}{\left(C_{i}C_{i}^{T}\right)}^{-1}\;C_{i}^{⊥}\right], \end{array}$$

where $C_{i}^{⊥}$ is called an orthogonal basis for the null space of *C*_*i*_ and $C_{i}C_{i}^{⊥}=0$.

Some essential lemmas are introduced to facilitate stability analysis.

Lemma 1 (Boyd et al., 1994). (Schur Complement) The appropriate dimensional matrices *S*_1,1_, *S*_1,2_, *S*_2,1_, and*S*_2,2_ satisfy $S_{1,1}=S_{1,1}^{T}$, $S_{1,2}=S_{2,1}^{T}$, and $S_{2,2}=S_{2,2}^{T}$such that the following conditions are equivalent:


$$\begin{array}{l} S=\left[\begin{array}{cc} S_{1,1} & S_{1,2}\\ S_{2,1} & S_{2,2} \end{array}\right] \end{array}$$

1) *S* < 0,

2) *S*_1,1_ < 0 and $S_{2,2}-S_{1,2}^{T}S_{1,1}^{-1}S_{1,2}<0$, and

3)*S*_2,2_ < 0 and $S_{1,1}-S_{1,2}S_{2,2}^{-1}S_{1,2}^{T}<0$.

Lemma 2 (Petersen, 1987). Let *P* = *P*^*T*^, *M*, and *N* be real matrices of appropriate dimensions. Then, *P* + *MF*(*t*)*N* + *N*^*T*^*F*^*T*^(*t*)*M*^*T*^ < 0 for all variable matrix functions *F*(*t*) satisfying *F*^*T*^(*t*)*F*(*t*) ≤ *I* if and only if there is a scalar ε > 0such that the following inequality holds:


$$\begin{array}{l} P+\varepsilon MM^{T}+\varepsilon^{-1}N^{T}N<0. \end{array}$$

Lemma 3 (Gahinet and Apkarian, 1994). (Finsler's lemma) The following conditions are equivalent:

1) *x*^*T*^ Ω *x* < 0, ∀*Wx* = 0, and *x* ≠ 0, where *x* is an augmented state vector;

2) *W*^⊥^*T* Ω *W*^⊥^ < 0, where *W*^⊥^is any null space basis matrix for *W*;

3) there is a scalar μ that satisfies Ω − μ*W*^*T*^*W* < 0; and

4) there is a matrix *X* that satisfies Ω + *XW* + *W*^*T*^*X*^*T*^ < 0.

Remark 2. $\sum_{k=1}^{r_{e}}v_{k}\left(\xi \left(t\right)\right)E_{k}$is required to be nonsingular to ensure that the augmented systems (2) maintain the impulse-free and regularization properties of the original system.

Remark 3. In this study, a nonlinear mechanical system is represented as a T-S fuzzy descriptor system instead of a T-S fuzzy system to effectively avoid the artificial introduction of different input matrices. By setting the system matrix *E*_*k*_ = *I*, a T-S fuzzy descriptor system can be transformed into a normal T-S fuzzy system. Therefore, the proposed controller is also feasible for a normal T-S fuzzy system.

## Non-Fragile Observer-Based Ismc For T-S Fuzzy Descriptor Systems

**Figure 1:**
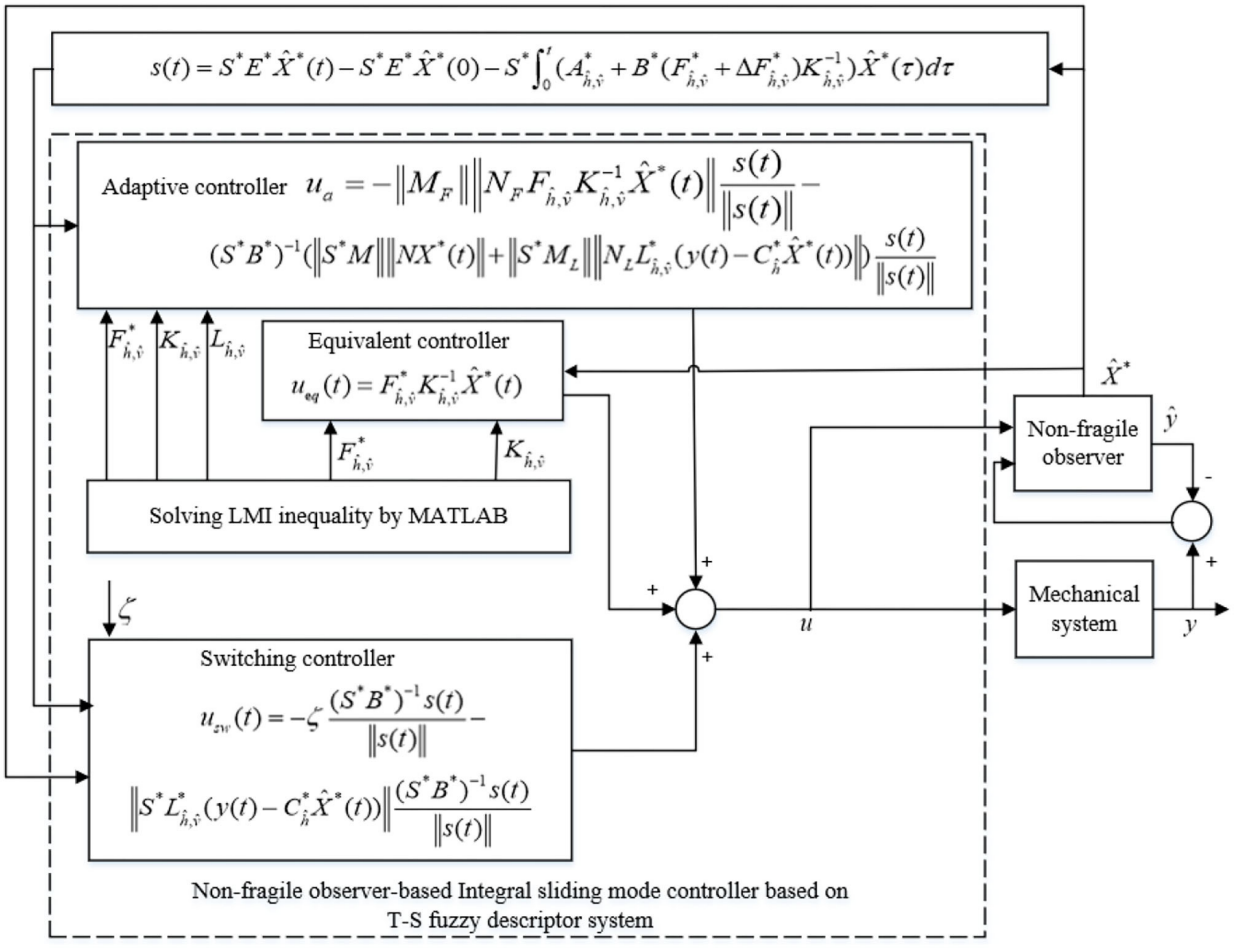
Non-fragile observer-based ISMC for T-S fuzzy descriptor systems.

When uncertainties of robotic system are considered such that ΔA^*^ ≠ 0, a robust observer is constructed to estimate the system states. In contrast, a non-fragile observer-based adaptive integral sliding mode controller for T-S fuzzy descriptor systems, as shown in Figure 1, is designed to address robotic system uncertainties and perturbations for both observer and controller structures. The control system includes two parts: a non-fragile observer and a non-fragile adaptive integral sliding mode controller. Moreover, the non-fragile adaptive integral sliding mode controller has three parts, namely, the equivalent control strategy, the switching control strategy, and the adaptive control strategy. The equivalent control strategy guarantees system convergence to the designed sliding surface, and the switching control strategy makes the closed-loop control system asymptotically stable. System uncertainties and perturbations associated with controller and observer gains are compensated for by the adaptive control strategy.

### Structure of the Non-Fragile Sliding Mode Observer

To estimate the states of system (2), the following non-fragile observer for estimating system states is considered:


(3) $$\begin{array}{l} E^{*}{\overset{∙}{\hat{X}}}^{*}\left(t\right)=\sum_{k=1}^{r_{e}}\\ \sum_{i}^{r}v_{k}\left(\hat{\xi }\left(t\right)\right)h_{i}\left(\hat{z}\left(t\right)\right)\left\{A_{k,i}^{*}{\hat{X}}^{*}\left(t\right)+B^{*}u\left(t\right)+\left(L_{k,i}^{*}+\text{Δ}L_{k,i}^{*}\right)\left(y\left(t\right)-\hat{y}\left(t\right)\right)\right\}, \end{array}$$

$$\begin{array}{l} \hat{y}\left(t\right)=\sum_{i=1}^{r}h_{i}\left(\hat{z}\left(t\right)\right)C_{i}^{*}{\hat{X}}^{*}\left(t\right), \end{array}$$

where ${\hat{X}}^{*}\left(t\right)$ is the state estimate of X^*^(*t*), *ŷ*(*t*) is the estimated value of the system output vector *y*(*t*), and $\hat{\xi }\left(t\right)$and*ẑ*(*t*)denote the estimated values of the premise variablesξ(*t*) and *z*(*t*), respectively. Using the notation presented in Introduction, Equation (3) can be represented as:


(4) $$\begin{array}{l} E^{*}{\overset{∙}{\hat{X}}}^{*}\left(t\right)=A_{\hat{h},\hat{v}}^{*}{\hat{X}}^{*}\left(t\right)+B^{*}u\left(t\right)+\left(L_{\hat{h},\hat{v}}^{*}+\text{Δ}L_{\hat{h},\hat{v}}^{*}\right)\left(y\left(t\right)-\hat{y}\left(t\right)\right), \end{array}$$

$$\begin{array}{l} \hat{y}\left(t\right)=C_{\hat{h}}^{*}{\hat{X}}^{*}\left(t\right), \end{array}$$

where $L_{\hat{h},\hat{v}}^{*}={\left[\begin{array}{cc} 0 & L_{\hat{h},\hat{v}}^{T} \end{array}\right]}^{T}$ and $\text{Δ}L_{\hat{h},\hat{v}}^{*}={\left[\begin{array}{cc} 0 & \text{Δ}L_{\hat{h},\hat{v}}^{T} \end{array}\right]}^{T}$. $L_{\hat{h},\hat{v}}$ denotes the observer gain, which will be determined later. $\text{Δ}L_{\hat{h},\hat{v}}=M_{L}S_{L}N_{L}L_{\hat{h},\hat{v}}$ is the observer gain perturbation, where *M*_*L*_ and *N*_*L*_ are known real constant matrices.*S*_*L*_ is an unknown time-varying matrix that satisfies $S_{L}^{T}S_{L}\leq I$.$\text{Δ}L_{\hat{h},\hat{v}}^{*}$ satisfies the following norm-bounded multiplicative relation:


(5) $$\begin{array}{l} \text{Δ}L_{\hat{h},\hat{v}}^{*}=M_{L}^{*}S_{L}N_{L}^{*}L_{\hat{h},\hat{v}}^{*}, \end{array}$$

where $M_{L}^{*}={\left[\begin{array}{cc} 0 & M_{L}^{T} \end{array}\right]}^{T}$ and $N_{L}^{*}=\left[\begin{array}{cc} 0 & N_{L} \end{array}\right]$.

The system state estimation error is defined as $e\left(t\right)=X^{*}\left(t\right)-{\hat{X}}^{*}\left(t\right)$; by considering the T-S fuzzy descriptor system (2) and the non-fragile observer system (4), the estimation error dynamic is obtained as:


(6) $$\begin{array}{l} E^{*}\dot{e}\left(t\right)=\left(A_{h,v}^{*}-\left(L_{\hat{h},\hat{v}}^{*}+\text{Δ}L_{\hat{h},\hat{v}}^{*}\right)C_{h}^{*}\right)e\left(t\right)\\ \;+\left(A_{h,v}^{*}-A_{\hat{h},\hat{v}}^{*}\right){\hat{X}}^{*}\left(t\right)\\ \;-\left(L_{\hat{h},\hat{v}}^{*}+\text{Δ}L_{\hat{h},\hat{v}}^{*}\right)\left(C_{h}^{*}-C_{\hat{h}}^{*}\right){\hat{X}}^{*}\left(t\right)\\ \;+\text{Δ}A^{*}X^{*}\left(t\right). \end{array}$$

Remark 4. Since we consider unmeasurable premise variables for T-S fuzzy descriptor systems, the membership functions of the T-S fuzzy descriptor system (*v*_*k*_(ξ(*t*)) and *h*_*i*_(*z*(*t*))) should be allowed to depend on the estimated system state ${\hat{x}}_{i}\left(t\right)$ rather than the original system state *x*_*i*_(*t*). Furthermore, the system output matrix *C*_*i*_(*t*) is allowed to be a function of the system state *x*_*i*_(*t*) instead of a constant matrix.

### Construction of the Integral Sliding Surface

Based on the non-fragile observer (4) and ISMC theory, the integral sliding surface function is designed as


(7) $$\begin{array}{l} s_{{\hat{X}}^{*}}\left(t\right)=S^{*}E^{*}{\hat{X}}^{*}\left(t\right)-S^{*}E^{*}{\hat{X}}^{*}\left(0\right) \end{array}$$

$$\begin{array}{ll} - & S^{*}\overset{t}{\underset{0}{\int }}\left(A_{\hat{h},\hat{v}}^{*}+B^{*}\left(F_{\hat{h},\hat{v}}^{*}+\text{Δ}F_{\hat{h},\hat{v}}^{*}\right)K_{\hat{h},\hat{v}}^{-1}\right){\hat{X}}^{*}\left(\tau \right)d\tau , \end{array}$$

where $F_{\hat{h},\hat{v}}^{*}=\left[\begin{array}{cc} F_{\hat{h},\hat{v}} & 0 \end{array}\right]$,$K_{\hat{h},\hat{v}}=\left[\begin{array}{cc} K_{1,\hat{v}} & K_{2,\hat{v}}\\ K_{3,\hat{h}} & K_{4,\hat{h}} \end{array}\right]$,$F_{\hat{h},\hat{v}}\in R^{m\times n}$, $K_{1,\hat{v}}\in R^{n\times n}$, $K_{2,\hat{v}}\in R^{n\times n}$, $K_{3,\hat{h}}\in R^{n\times n}$, and $K_{4,\hat{h}}\in R^{n\times n}$ are the system controller gains, which will be determined later. S^*^ is a constant matrix that satisfies det(S^*^B^*^) ≠ 0. $\text{Δ}F_{\hat{h},\hat{v}}^{*}=\left[\text{Δ}\begin{array}{cc} F_{\hat{h},\hat{v}} & 0 \end{array}\right]$is the controller gain perturbation and satisfies the following norm-bounded multiplicative relation:


(8) $$\begin{array}{l} \text{Δ}F_{\hat{h},\hat{v}}^{*}=M_{F}S_{F}N_{F}F_{\hat{h},\hat{v}}^{*}, \end{array}$$

where *M*_*F*_ and *N*_*F*_ are known matrices with appropriate dimensions, and *S*_*F*_ is an unknown time-varying matrix that satisfies $S_{F}^{T}S_{F}\leq I$.

Combining (4) with (7), the derivative of the integral sliding mode surface (7) can be obtained as:


(9) $$\begin{array}{l} {\dot{s}}_{{\hat{X}}^{*}}\left(t\right)=\\ S^{*}\left(A_{\hat{h},\hat{v}}^{*}{\hat{X}}^{*}\left(t\right)+B^{*}u\left(t\right)+\left(L_{\hat{h},\hat{v}}^{*}+\text{Δ}L_{\hat{h},\hat{v}}^{*}\right)\left(y\left(t\right)-C_{\hat{h}}^{*}{\hat{X}}^{*}\left(t\right)\right)\right)\\ -S^{*}\left(A_{\hat{h},\hat{v}}^{*}+B^{*}\left(F_{\hat{h},\hat{v}}^{*}+\text{Δ}F_{\hat{h},\hat{v}}^{*}\right)K_{\hat{h},\hat{v}}^{-1}\right){\hat{X}}^{*}\left(t\right). \end{array}$$

When system trajectories reach the ideal sliding surface, the following conditions must be satisfied: $s_{{\hat{X}}^{*}}\left(t\right)=0$ and ${\dot{s}}_{{\hat{X}}^{*}}\left(t\right)=0$. Therefore, the equivalent control can be designed as follows:

Case 1:$C_{h}=C_{\hat{h}}=C^{*}$


(10) $$\begin{array}{l} u_{eq}\left(t\right)=\left(F_{\hat{h},\hat{v}}^{*}+\text{Δ}F_{\hat{h},\hat{v}}^{*}\right)K_{\hat{h},\hat{v}}^{-1}{\hat{X}}^{*}\left(t\right)\\ \;-{\left(S^{*}B^{*}\right)}^{-1}S^{*}\left(L_{\hat{h},\hat{v}}^{*}+\text{Δ}L_{\hat{h},\hat{v}}^{*}\right)C^{*}e\left(t\right). \end{array}$$

Case 2:*C*_*h*_ ≠ *C*_$\hat{h}$_


(11) $$\begin{array}{l} u_{eq}\left(t\right)=\left(F_{\hat{h},\hat{v}}^{*}+\text{Δ}F_{\hat{h},\hat{v}}^{*}\right)K_{\hat{h},\hat{v}}^{-1}{\hat{X}}^{*}\left(t\right)\\ \;-{\left(S^{*}B^{*}\right)}^{-1}S^{*}\left(L_{\hat{h},\hat{v}}^{*}+\text{Δ}L_{\hat{h},\hat{v}}^{*}\right)C_{h}^{*}e\left(t\right)\\ \;-{\left(S^{*}B^{*}\right)}^{-1}S^{*}\left(L_{\hat{h},\hat{v}}^{*}+\text{Δ}L_{\hat{h},\hat{v}}^{*}\right)\left(C_{h}^{*}-C_{\hat{h}}^{*}\right){\hat{X}}^{*}\left(t\right). \end{array}$$

By substituting the equivalent controller (10-11) into the sliding mode observer system (4), the sliding mode dynamics are established as (12) and (14). Meanwhile, the dynamic estimation error Equation (6) is redescribed as (13) and (15).

Case 1:$C_{h}=C_{\hat{h}}=C^{*}$


(12) $$\begin{array}{l} E^{*}{\overset{∙}{\hat{X}}}^{*}\left(t\right)=\left(A_{\hat{h},\hat{v}}^{*}+B^{*}\left(F_{\hat{h},\hat{v}}^{*}+\text{Δ}F_{\hat{h},\hat{v}}^{*}\right)K_{\hat{h},\hat{v}}^{-1}\right){\hat{X}}^{*}\left(t\right)\\ \;+\bar{BB}\left(L_{\hat{h},\hat{v}}^{*}+\text{Δ}L_{\hat{h},\hat{v}}^{*}\right)C^{*}e\left(t\right), \end{array}$$

(13) $$\begin{array}{l} E^{*}\dot{e}\left(t\right)=\left(A_{h,v}^{*}-\left(L_{\hat{h},\hat{v}}^{*}+\text{Δ}L_{\hat{h},\hat{v}}^{*}\right)C^{*}+\text{Δ}A^{*}\right)e\left(t\right)+\left(A_{h,v}^{*}-A_{\hat{h},\hat{v}}^{*}+\text{Δ}A^{*}\right){\hat{X}}^{*}\left(t\right). \end{array}$$

Case 2:*C*_*h*_ ≠ *C*_$\hat{h}$_


(14) $$\begin{array}{l} E^{*}{\overset{∙}{\hat{X}}}^{*}\left(t\right)=\left(A_{\hat{h},\hat{v}}^{*}+B^{*}\left(F_{\hat{h},\hat{v}}^{*}+\text{Δ}F_{\hat{h},\hat{v}}^{*}\right)K_{\hat{h},\hat{v}}^{-1}+\bar{BB}\left(L_{\hat{h},\hat{v}}^{*}+\text{Δ}L_{\hat{h},\hat{v}}^{*}\right)\left(C_{h}^{*}-C_{\hat{h}}^{*}\right)\right){\hat{X}}^{*}\left(t\right)+\bar{BB}\left(L_{\hat{h},\hat{v}}^{*}+\text{Δ}L_{\hat{h},\hat{v}}^{*}\right)C_{h}^{*}e\left(t\right), \end{array}$$

(15) $$\begin{array}{l} E^{*}\dot{e}\left(t\right)=\left(A_{h,v}^{*}-\left(L_{\hat{h},\hat{v}}^{*}+\text{Δ}L_{\hat{h},\hat{v}}^{*}\right)C_{h}^{*}+\text{Δ}A^{*}\right)e\left(t\right)+\left(A_{h,v}^{*}-A_{\hat{h},\hat{v}}^{*}-\left(L_{\hat{h},\hat{v}}^{*}+\text{Δ}L_{\hat{h},\hat{v}}^{*}\right)\left(C_{h}^{*}-C_{\hat{h}}^{*}\right)+\text{Δ}A^{*}\right){\hat{X}}^{*}\left(t\right), \end{array}$$

where $\bar{BB}=I-B^{*}{\left(S^{*}B^{*}\right)}^{-1}S^{*}$. In this study, the control goal is to obtain the observer gains and controller gains such that ${\hat{X}}^{*}\left(t\right)\to 0$ and *e*(*t*) → 0 for *t* → ∞.

Equations (12) and (14) can be rearranged as follows:

Case 1:$C_{h}=C_{\hat{h}}=C^{*}$


(16) $$\begin{array}{l} \left[\begin{array}{ccc} A_{\hat{h},\hat{v}}^{*}+B^{*}\left(F_{\hat{h},\hat{v}}^{*}+\text{Δ}F_{\hat{h},\hat{v}}^{*}\right)K_{\hat{h},\hat{v}}^{-1} & -I & \bar{BB}\left(L_{\hat{h},\hat{v}}^{*}+\text{Δ}L_{\hat{h},\hat{v}}^{*}\right)C^{*} \end{array}\right]\left[\begin{array}{c} {\hat{X}}^{*}\left(t\right)\\ E^{*}{\overset{∙}{\hat{X}}}^{*}V\left(t\right)\\ e\left(t\right) \end{array}\right]=0. \end{array}$$

Case 2:*C*_*h*_ ≠ *C*_$\hat{h}$_


(17) $$\begin{array}{l} \left[\begin{array}{ccc} A_{\hat{h},\hat{v}}^{*}+B^{*}\left(F_{\hat{h},\hat{v}}^{*}+\text{Δ}F_{\hat{h},\hat{v}}^{*}\right)K_{\hat{h},\hat{v}}^{-1}+\bar{BB}\left(L_{\hat{h},\hat{v}}^{*}+\text{Δ}L_{\hat{h},\hat{v}}^{*}\right)\left(C_{h}^{*}-C_{\hat{h}}^{*}\right) & -I & \bar{BB}\left(L_{\hat{h},\hat{v}}^{*}+\text{Δ}L_{\hat{h},\hat{v}}^{*}\right)C_{h}^{*} \end{array}\right]\left[\begin{array}{c} {\hat{X}}^{*}\left(t\right)\\ E^{*}{\overset{∙}{\hat{X}}}^{*}\left(t\right)\\ e\left(t\right) \end{array}\right]=0. \end{array}$$

### Admissibility Analysis of the Dynamic Sliding Mode System

Based on the LMI theory and the Lyapunov stability theory, the controller gains and observer gains are determined in this section such that sliding mode dynamics are asymptotically stable.

Theorem 1: Suppose that the T-S fuzzy descriptor system matrices in (2) satisfy ΔA^*^ ≠ 0 and $C_{h}=C_{\hat{h}}=C^{*}$. Given constants ε > 0 and ε_1_ > 0, the closed-loop systems (12,13) are asymptotically stable if positive definite matrices *P*_1_ and *Q*_1_, scalars ε_2,*i,k*_, $\varepsilon_{\text{3,}i,k}^{E}$, $\varepsilon_{\text{3,}i,k}^{A}$, and ε_4,*i,k*_, and a set of matrices *P*_3,*i,k*_, *P*_4,*i,k*_, *F*_*i,k*_, *K*_1,*k*_, *K*_2,*k*_, *K*_3,*i*_, *K*_4,*i*_, *L*_*i,k*_, *R*_1_, $R_{1,i,k}^{11}$, $R_{1,i,k}^{12}$, $R_{1,i,k}^{2}$, $R_{1,i,k}^{3}$, and $R_{1,i,k}^{4}$ exist, where *k* ∈ {1, ⋯ , *r*_*e*_} and *i* ∈ {1, ⋯ , *r*}, such that the following LMIs hold:


(18) $$\begin{array}{l} \left[\begin{array}{cc} {\text{ Ω }}_{1} & *\\ {\text{ Ω }}_{2} & {\text{ Ω }}_{3} \end{array}\right]<0, \end{array}$$

$$\begin{array}{c} {\text{ Ω }}_{1}=\left[\begin{array}{cccccccc} {\text{Δ}}_{1,1} & * & * & * & * & * & * & *\\ {\text{Δ}}_{2,1} & {\text{Δ}}_{2,2} & * & * & * & * & * & *\\ {\text{Δ}}_{3,1} & {\text{Δ}}_{3,2} & -\varepsilon \text{He(}P_{1}\text{)} & * & * & * & * & *\\ {\text{Δ}}_{4,1} & {\text{Δ}}_{4,2} & -\varepsilon P_{3,\hat{h},\hat{v}} & {\text{Δ}}_{4,4} & * & * & * & *\\ 0 & {\left(\bar{B}Y_{\hat{h},\hat{v}}\right)}^{T} & 0 & \varepsilon {\left(\bar{B}Y_{\hat{h},\hat{v}}\right)}^{T} & \text{He(}Q_{3,\hat{h}}\text{)} & * & * & *\\ {\text{Δ}}_{6,1} & {\text{Δ}}_{6,2} & 0 & -\varepsilon M_{L}{\;\varepsilon \;}_{4,\hat{h},\hat{v}}M_{L}^{T}{\bar{B}}^{T} & {\text{Δ}}_{6,5} & {\text{Δ}}_{6,6} & * & *\\ 0 & \varepsilon_{1}{\left(\bar{B}Y_{\hat{h},\hat{v}}\right)}^{T} & 0 & \varepsilon_{1}\varepsilon {\left(\bar{B}Y_{\hat{h},\hat{v}}\right)}^{T} & Q_{1}-TR_{1,\hat{h},\hat{v}}^{1} & {\text{Δ}}_{7,6} & {\text{Δ}}_{7,7} & *\\ 0 & 0 & 0 & 0 & Q_{3,\hat{h}}-R_{1,\hat{h},\hat{v}}^{3} & {\text{Δ}}_{8,6} & {\text{Δ}}_{8,7} & -\varepsilon_{1}\text{He(}R_{1,\hat{h},\hat{v}}^{4}\text{)} \end{array}\right],\\ {\text{ Ω }}_{2}=\left[\begin{array}{cccccccc} N_{F}F_{\hat{h},\hat{v}} & 0 & 0 & 0 & 0 & 0 & 0 & 0\\ N_{A}K_{1,\hat{v}} & N_{A}K_{2,\hat{v}} & 0 & 0 & N_{A}Q_{1} & 0 & 0 & 0\\ N_{E}K_{3,\hat{h}} & N_{E}K_{4,\hat{h}} & 0 & 0 & N_{E}Q_{3,\hat{h}} & N_{E}Q_{4,\hat{h}} & 0 & 0\\ 0 & 0 & 0 & 0 & N_{L}Y_{\hat{h},\hat{v}} & 0 & \varepsilon_{1}N_{L}Y_{\hat{h},\hat{v}} & 0 \end{array}\right],\\ {\text{ Ω }}_{3}=\left[\begin{array}{cccc} -{\;\varepsilon \;}_{2,\hat{h},\hat{v}}I & * & * & *\\ 0 & -\;\varepsilon {\;}_{3,\hat{h},\hat{v}}^{A}I & * & *\\ 0 & 0 & -\;\varepsilon {\;}_{3,\hat{h},\hat{v}}^{E}I & *\\ 0 & 0 & 0 & -{\;\varepsilon \;}_{4,\hat{h},\hat{v}}I \end{array}\right], \end{array}$$

where Δ_1,1_ = He(*K*_3,$\hat{h}$_), ${\text{Δ}}_{2,1}=A_{\hat{h}}K_{1,\hat{v}}-E_{\hat{v}}K_{3,\hat{h}}+BF_{\hat{h},\hat{v}}+K_{4,\hat{h}}^{T}$, ${\text{Δ}}_{2,2}=\text{He(}A_{\hat{h}}K_{2,\hat{v}})+BM_{F}{\;\varepsilon \;}_{2,\hat{h},\hat{v}}M_{F}^{T}B^{T}-$$\text{He}(E_{\hat{v}}K_{4,\hat{h}}\text{)}+\bar{B}M_{L}{\;\varepsilon \;}_{4,\hat{h},\hat{v}}M_{L}^{T}{\bar{B}}^{T}$, ${\text{Δ}}_{3,1}=K_{1,\hat{v}}+\varepsilon K_{3,\hat{h}}-P_{1}$, ${\text{Δ}}_{3,2}=K_{2,\hat{v}}+\varepsilon K_{4,\hat{h}}$, ${\text{Δ}}_{4,1}=K_{3,\hat{h}}-P_{3,\hat{h},\hat{v}}+$$\varepsilon \left(A_{\hat{h}}K_{1,\hat{v}}-E_{\hat{v}}K_{3,\hat{h}}+BF_{\hat{h},\hat{v}}\right)$, ${\text{Δ}}_{4,2}=\varepsilon BM_{F}{\;\varepsilon \;}_{2,\hat{h},\hat{v}}M_{F}^{T}B^{T}+\varepsilon \bar{B}M_{L}{\;\varepsilon \;}_{4,\hat{h},\hat{v}}M_{L}^{T}{\bar{B}}^{T}+K_{4,\hat{h}}+\varepsilon A_{\hat{h}}K_{2,\hat{v}}-P_{4,\hat{h},\hat{v}}-$$\varepsilon E_{\hat{v}}K_{4,\hat{h}}$, ${\text{Δ}}_{4,4}=\varepsilon^{2}BM_{F}{\;\varepsilon \;}_{2,\hat{h},\hat{v}}M_{F}^{T}B^{T}+\varepsilon^{2}\bar{B}M_{L}{\;\varepsilon \;}_{4,\hat{h},\hat{v}}M_{L}^{T}{\bar{B}}^{T}-\varepsilon \text{He(}P_{4,\hat{h},\hat{v}}\text{)}$, ${\text{Δ}}_{6,1}=\left(A_{h}-A_{\hat{h}}\right)K_{1,\hat{v}}-$$E_{v}K_{3,\hat{h}}+E_{\hat{v}}K_{3,\hat{h}}$, ${\text{Δ}}_{6,2}=\left(A_{h}-A_{\hat{h}}\right)K_{2,\hat{v}}-\left(E_{v}-E_{\hat{v}}\right)K_{4,\hat{h}}-M_{L}{\;\varepsilon \;}_{4,\hat{h},\hat{v}}M_{L}^{T}{\bar{B}}^{T}$, ${\text{Δ}}_{6,5}=Q_{4,\hat{h}}^{T}-$ $Y_{\hat{h},\hat{v}}+A_{h}Q_{1}-$*E*_*v*_*Q*_3,$\hat{h}$_, ${\text{Δ}}_{6,6}=-\text{He(}E_{v}Q_{4,\hat{h}}\text{)}+M_{L}{\;\varepsilon \;}_{4,\hat{h},\hat{v}}M_{L}^{T}+M_{A}\;\varepsilon {\;}_{3,\hat{h},\hat{v}}^{A}M_{A}^{T}+M_{E}\;\varepsilon {\;}_{3,\hat{h},\hat{v}}^{E}M_{E}^{T}$, ${\text{Δ}}_{7,6}=-\varepsilon_{1}{\left(Y_{\hat{h},\hat{v}}\right)}^{T}-TR_{1,\hat{h},\hat{v}}^{2}$, ${\text{Δ}}_{7,7}=-\varepsilon_{1}\text{He(}TR_{1,\hat{h},\hat{v}}^{1}\text{)}$, ${\text{Δ}}_{8,6}=Q_{4,\hat{h}}-R_{1,\hat{h},\hat{v}}^{4}$, ${\text{Δ}}_{8,7}=-\varepsilon_{1}R_{1,\hat{h},\hat{v}}^{3}-\varepsilon_{1}{\left(TR_{1,\hat{h},\hat{v}}^{2}\right)}^{T}$, $\bar{B}=B{\left(S_{2}^{*}B\right)}^{-1}S_{2}^{*}$, $Y_{\hat{h},\hat{v}}=$$\left[\begin{array}{cc} {\left(L_{\hat{h},\hat{v}}R_{1}\right)}_{n\times p} & 0_{n\times \left(n-p\right)} \end{array}\right]$, $R_{1,\hat{h},\hat{v}}^{1}=\left[\begin{array}{cc} R_{1} & 0_{p\times \left(n-p\right)}\\ R_{1,\hat{h},\hat{v}}^{11} & R_{1,\hat{h},\hat{v}}^{12} \end{array}\right]$, and $R_{1,\hat{h},\hat{v}}^{2}=\left[\begin{array}{c} 0_{p\times n}\\ R_{1,\hat{h},\hat{v}}^{21} \end{array}\right]$.

Proof: The Lyapunov function candidate is constructed as:


(19) $$\begin{array}{l} \text{V}\left(\hat{x}\text{(}t\text{),}e_{1}\left(t\right)\right)={\hat{x}}^{T}\left(t\right)P_{1}^{-1}\hat{x}\left(t\right)+e_{1}^{T}\left(t\right)Q_{1}^{-1}e_{1}\left(t\right)={\hat{X}}^{*T}\left(t\right)E^{*T}P_{\hat{h},\hat{v}}^{-1}{\hat{X}}^{*}\left(t\right)+e^{T}\left(t\right)E^{*T}Q_{\hat{h},\hat{v}}^{-1}e\left(t\right), \end{array}$$

where $P_{\hat{h},\hat{v}}=\left[\begin{array}{cc} P_{1} & 0\\ P_{3,\hat{h},\hat{v}} & P_{4,\hat{h},\hat{v}} \end{array}\right]$, $P_{\hat{h},\hat{v}}^{-1}=\left[\begin{array}{cc} P_{1}^{-1} & 0\\ -P_{4,\hat{h},\hat{v}}^{-1}P_{3,\hat{h},\hat{v}}P_{1}^{-1} & P_{4,\hat{h},\hat{v}}^{-1} \end{array}\right]$, $P_{1}=P_{1}^{T}$, $Q_{\hat{h},\hat{v}}=\left[\begin{array}{cc} Q_{1} & 0\\ Q_{3,\hat{h},\hat{v}} & Q_{4,\hat{h},\hat{v}} \end{array}\right]$, $Q_{1}=Q_{1}^{T}$, $P_{1}\in R^{n\times n}$, $P_{3,\hat{h},\hat{v}}\in R^{n\times n}$, $P_{4,\hat{h},\hat{v}}\in R^{n\times n}$, $Q_{1}\in R^{n\times n}$, $Q_{3,\hat{h},\hat{v}}\in R^{n\times n}$, $Q_{4,\hat{h},\hat{v}}\in R^{n\times n}$, and $e_{1}\left(t\right)=x\left(t\right)-\hat{x}\left(t\right)$.The matrix and its derivative satisfy $E^{*T}P_{\hat{h},\hat{v}}^{-1}=P_{\hat{h},\hat{v}}^{-T}E^{*}=\left[\begin{array}{cc} P_{1} & 0\\ 0 & 0 \end{array}\right]$, $E^{*T}Q_{\hat{h},\hat{v}}^{-1}=Q_{\hat{h},\hat{v}}^{-T}E^{*}=\left[\begin{array}{cc} Q_{1} & 0\\ 0 & 0 \end{array}\right]$, $\frac{d}{dt}\left(E^{*T}P_{\hat{h},\hat{v}}^{-1}\right)=0$, and $\frac{d}{dt}\left(E^{*T}Q_{\hat{h},\hat{v}}^{-1}\right)=0$. Therefore, the derivative of the Lyapunov function (19) is calculated as:


(20) $$\begin{array}{l} \overset{∙}{V}\left(\hat{x}\left(t\right),e_{1}\left(t\right)\right)={\overset{∙}{\hat{X}}}^{*T}\left(t\right)E^{*T}P_{\hat{h},\hat{v}}^{-1}{\hat{X}}^{*}\left(t\right)+{\hat{X}}^{*T}\left(t\right)P_{\hat{h},\hat{v}}^{-T}E^{*}{\overset{∙}{\hat{X}}}^{*}\left(t\right)+{\dot{e}}^{T}\left(t\right)E^{*T}Q_{\hat{h},\hat{v}}^{-1}e\left(t\right)+e^{T}\left(t\right)Q_{\hat{h},\hat{v}}^{-T}E^{*}\dot{e}\left(t\right) \end{array}$$

$$\begin{array}{l} ={\left[\begin{array}{c} {\hat{X}}^{*}\left(t\right)\\ E^{*}{\overset{∙}{\hat{X}}}^{*}\left(t\right)\\ e\left(t\right) \end{array}\right]}^{T}\left[\begin{array}{ccc} 0 & * & *\\ P_{\hat{h},\hat{v}}^{-1} & 0 & *\\ Q_{\hat{h},\hat{v}}^{-T}\left(A_{h,v}^{*}-A_{\hat{h},\hat{v}}^{*}+\text{Δ}A^{*}\right) & 0 & \text{He((}A_{h,v}^{*}-\left(L_{\hat{h},\hat{v}}^{*}+\text{Δ}L_{\hat{h},\hat{v}}^{*}\right)C^{*}+\text{Δ}A^{*}{\text{)}}^{T}Q_{\hat{h},\hat{v}}^{-1}\text{)} \end{array}\right]\left[\begin{array}{c} {\hat{X}}^{*}\left(t\right)\\ E^{*}{\overset{∙}{\hat{X}}}^{*}\left(t\right)\\ e\left(t\right) \end{array}\right]<0, \end{array}$$

If (20) holds, then the following inequality can be obtained by Finsler's lemma (lemma 3) under the constraint in the sliding mode dynamics Equation (16):


(21) $$\begin{array}{l} \left[\begin{array}{ccc} 0 & * & *\\ P_{\hat{h},\hat{v}}^{-1} & 0 & *\\ Q_{\hat{h},\hat{v}}^{-T}\left(A_{h,v}^{*}-A_{\hat{h},\hat{v}}^{*}+\text{Δ}A^{*}\right) & 0 & \text{He((}A_{h,v}^{*}-\left(L_{\hat{h},\hat{v}}^{*}+\text{Δ}L_{\hat{h},\hat{v}}^{*}\right)C^{*}+\text{Δ}A^{*}{\text{)}}^{T}Q_{\hat{h},\hat{v}}^{-1}\text{)} \end{array}\right]+{\text{Ξ}}_{1}<0,\\ {\text{Ξ}}_{1}\text{= He}\left(\left[\begin{array}{c} U_{\hat{h},\hat{v}}\\ V_{\hat{h},\hat{v}}\\ W_{\hat{h},\hat{v}} \end{array}\right]\left[\begin{array}{ccc} A_{\hat{h},\hat{v}}^{*}+B^{*}\left(F_{\hat{h},\hat{v}}^{*}+\text{Δ}F_{\hat{h},\hat{v}}^{*}\right)K_{\hat{h},\hat{v}}^{-1} & -I & \bar{BB}\left(L_{\hat{h},\hat{v}}^{*}+\text{Δ}L_{\hat{h},\hat{v}}^{*}\right)C^{*} \end{array}\right]\right), \end{array}$$

where $U_{\hat{h},\hat{v}}$, $V_{\hat{h},\hat{v}}$, and $W_{\hat{h},\hat{v}}$ are matrix variables with appropriate dimensions. By pre- and post-multiplying (21) by $\left[\begin{array}{ccc} K_{\hat{h},\hat{v}}^{T} & 0 & 0\\ 0 & P_{\hat{h},\hat{v}} & 0\\ 0 & 0 & Q_{\hat{h},\hat{v}}^{T} \end{array}\right]$ and $\left[\begin{array}{ccc} K_{\hat{h},\hat{v}} & 0 & 0\\ 0 & P_{\hat{h},\hat{v}}^{T} & 0\\ 0 & 0 & Q_{\hat{h},\hat{v}} \end{array}\right]$, respectively, the following inequality is obtained:


(22) $$\begin{array}{l} \left[\begin{array}{ccc} 0 & * & *\\ K_{\hat{h},\hat{v}} & 0 & *\\ \left(A_{h,v}^{*}-A_{\hat{h},\hat{v}}^{*}+\text{Δ}A^{*}\right)K_{\hat{h},\hat{v}} & 0 & \text{He(}Q_{\hat{h},\hat{v}}^{T}{\left(A_{h,v}^{*}-\left(L_{\hat{h},\hat{v}}^{*}+\text{Δ}L_{\hat{h},\hat{v}}^{*}\right)C^{*}+\text{Δ}A^{*}\right)}^{T}\text{)} \end{array}\right]+{\text{Ξ}}_{2}<0, \end{array}$$

$$\begin{array}{l} {\text{Ξ}}_{2}\text{=He(}\left[\begin{array}{c} K_{\hat{h},\hat{v}}^{T}U_{\hat{h},\hat{v}}\\ P_{\hat{h},\hat{v}}V_{\hat{h},\hat{v}}\\ Q_{\hat{h},\hat{v}}^{T}W_{\hat{h},\hat{v}} \end{array}\right]\left[\begin{array}{ccc} A_{\hat{h},\hat{v}}^{*}K_{\hat{h},\hat{v}}+B^{*}\left(F_{\hat{h},\hat{v}}^{*}+\text{Δ}F_{\hat{h},\hat{v}}^{*}\right) & -P_{\hat{h},\hat{v}}^{T} & \bar{BB}\left(L_{\hat{h},\hat{v}}^{*}+\text{Δ}L_{\hat{h},\hat{v}}^{*}\right)C^{*}Q_{\hat{h},\hat{v}} \end{array}\right]\text{)} \end{array}$$

where $U_{\hat{h},\hat{v}}=K_{\hat{h},\hat{v}}^{-T}$, $V_{\hat{h},\hat{v}}=\varepsilon P_{\hat{h},\hat{v}}^{-1}$, and $W_{\hat{h},\hat{v}}=0$ are defined for ε > 0. Then, inequality (22) can be represented as:


(23) $$\begin{array}{l} {\text{Ξ}}_{3}+\text{He(}\left[\begin{array}{ccc} 0 & 0 & 0\\ 0 & 0 & 0\\ {\left(\bar{BB}\left(L_{\hat{h},\hat{v}}^{*}+\text{Δ}L_{\hat{h},\hat{v}}^{*}\right)C^{*}Q_{\hat{h},\hat{v}}\right)}^{T} & \varepsilon {\left(\bar{BB}\left(L_{\hat{h},\hat{v}}^{*}+\text{Δ}L_{\hat{h},\hat{v}}^{*}\right)C^{*}Q_{\hat{h},\hat{v}}\right)}^{T} & -{\left(\left(L_{\hat{h},\hat{v}}^{*}+\text{Δ}L_{\hat{h},\hat{v}}^{*}\right)C^{*}Q_{\hat{h},\hat{v}}\right)}^{T} \end{array}\right]\text{) <0}, \end{array}$$

$$\begin{array}{l} {\text{ Ξ }}_{3}=\left[\begin{array}{ccc} \text{He(}A_{\hat{h},\hat{v}}^{*}K_{\hat{h},\hat{v}}+B^{*}\left(F_{\hat{h},\hat{v}}^{*}+\text{Δ}F_{\hat{h},\hat{v}}^{*}\right)\text{)} & * & *\\ K_{\hat{h},\hat{v}}-P_{\hat{h},\hat{v}}+\varepsilon \left(A_{\hat{h},\hat{v}}^{*}K_{\hat{h},\hat{v}}+B^{*}\left(F_{\hat{h},\hat{v}}^{*}+\text{Δ}F_{\hat{h},\hat{v}}^{*}\right)\right) & -\varepsilon \text{He(}P_{\hat{h},\hat{v}}^{T}\text{)} & *\\ \left(A_{h,v}^{*}-A_{\hat{h},\hat{v}}^{*}\right)K_{\hat{h},\hat{v}}+\text{Δ}A^{*}K_{\hat{h},\hat{v}} & 0 & \text{He(Δ}A^{*}Q_{\hat{h},\hat{v}}+A_{h,v}^{*}Q_{\hat{h},\hat{v}}\text{)} \end{array}\right], \end{array}$$

The corresponding auxiliary variables are introduced into the system, and the dimension of the system is increased with the augmented method to eliminate the coupling of the Lyapunov function matrix $Q_{\hat{h},\hat{v}}$, the observer gain matrix $L_{\hat{h},\hat{v}}^{*}$, and the observer gain perturbation $\text{Δ}L_{\hat{h},\hat{v}}^{*}$ in inequality (23). The following equations are defined:


(24) $$\begin{array}{l} x_{4}\left(t\right)={\left(\bar{BB}\left(L_{\hat{h},\hat{v}}^{*}+\text{Δ}L_{\hat{h},\hat{v}}^{*}\right)C^{*}\right)}^{T}x_{1}\left(t\right)+\varepsilon {\left(\bar{BB}\left(L_{\hat{h},\hat{v}}^{*}+\text{Δ}L_{\hat{h},\hat{v}}^{*}\right)C^{*}\right)}^{T}x_{2}\left(t\right)-{\left(\left(L_{\hat{h},\hat{v}}^{*}+\text{Δ}L_{\hat{h},\hat{v}}^{*}\right)C^{*}\right)}^{T}x_{3}\left(t\right). \end{array}$$

Thus, inequality (23) can be expressed as:


(25) $$\begin{array}{l} {\left[\begin{array}{c} x_{1}\left(t\right)\\ x_{2}\left(t\right)\\ x_{3}\left(t\right)\\ x_{4}\left(t\right) \end{array}\right]}^{T}{\text{ Ξ }}_{4}\left[\begin{array}{c} x_{1}\left(t\right)\\ x_{2}\left(t\right)\\ x_{3}\left(t\right)\\ x_{4}\left(t\right) \end{array}\right]<0, \end{array}$$

$$\begin{array}{l} {\text{ Ξ }}_{4}=\left[\begin{array}{cccc} \text{He(}A_{\hat{h},\hat{v}}^{*}K_{\hat{h},\hat{v}}+B^{*}\left(F_{\hat{h},\hat{v}}^{*}+\text{Δ}F_{\hat{h},\hat{v}}^{*}\right)\text{)} & * & * & *\\ \text{ Ξ}{\;}_{4}^{1} & -\varepsilon \text{He(}P_{\hat{h},\hat{v}}^{T}\text{)} & * & *\\ \left(A_{h,v}^{*}-A_{\hat{h},\hat{v}}^{*}\right)K_{\hat{h},\hat{v}}+\text{Δ}A^{*}K_{\hat{h},\hat{v}} & 0 & \text{He(Δ}A^{*}Q_{\hat{h},\hat{v}}+A_{h,v}^{*}Q_{\hat{h},\hat{v}}\text{)} & *\\ 0 & 0 & Q_{\hat{h},\hat{v}} & 0 \end{array}\right] \end{array}$$

where $\text{ Ξ}{\;}_{4}^{1}=K_{\hat{h},\hat{v}}+\varepsilon \left(A_{\hat{h},\hat{v}}^{*}K_{\hat{h},\hat{v}}+B^{*}\left(F_{\hat{h},\hat{v}}^{*}+\text{Δ}F_{\hat{h},\hat{v}}^{*}\right)\right)-P_{\hat{h},\hat{v}}$.

If (25) holds, then the following inequality can be obtained by Finsler's lemma (lemma 3) under the constraint in Equation (24).


(26) $$\begin{array}{l} {\text{ Ξ }}_{4}+{\text{ Ξ }}_{5}<0, \end{array}$$

$$\begin{array}{l} {\text{ Ξ }}_{5}=\text{He(}\left[\begin{array}{c} 0\\ 0\\ {\left(T^{*}R_{1,\hat{h},\hat{v}}\right)}^{T}\\ \varepsilon_{1}{\left(T^{*}R_{1,\hat{h},\hat{v}}\right)}^{T} \end{array}\right]\left[\begin{array}{cccc} {\left(\bar{BB}\left(L_{\hat{h},\hat{v}}^{*}+\text{Δ}L_{\hat{h},\hat{v}}^{*}\right)C^{*}\right)}^{T} & \varepsilon {\left(\bar{BB}\left(L_{\hat{h},\hat{v}}^{*}+\text{Δ}L_{\hat{h},\hat{v}}^{*}\right)C^{*}\right)}^{T} & -{\left(\left(L_{\hat{h},\hat{v}}^{*}+\text{Δ}L_{\hat{h},\hat{v}}^{*}\right)C^{*}\right)}^{T} & -I \end{array}\right]\text{)} \end{array}$$

where $R_{1,\hat{h},\hat{v}}=\left[\begin{array}{cc} R_{1,\hat{h},\hat{v}}^{1} & R_{1,\hat{h},\hat{v}}^{2}\\ R_{1,\hat{h},\hat{v}}^{3} & R_{1,\hat{h},\hat{v}}^{4} \end{array}\right]$, $R_{1,\hat{h},\hat{v}}^{1}=\left[\begin{array}{cc} R_{1} & 0_{p\times \left(n-p\right)}\\ R_{1,\hat{h},\hat{v}}^{11} & R_{1,\hat{h},\hat{v}}^{12} \end{array}\right]$, $R_{1,\hat{h},\hat{v}}^{2}=\left[\begin{array}{c} 0_{p\times n}\\ R_{1,\hat{h},\hat{v}}^{21} \end{array}\right]$, $T^{*}=\left[\begin{array}{cc} T & 0\\ 0 & I \end{array}\right]$,$R_{1}\in R^{p\times p}$, $R_{1,\hat{h},\hat{v}}^{11}\in R^{\left(n-p\right)\times p}$, $R_{1,\hat{h},\hat{v}}^{12}\in R^{\left(n-p\right)\times \left(n-p\right)}$, $R_{1,\hat{h},\hat{v}}^{21}\in R^{\left(n-p\right)\times n}$, $R_{1,\hat{h},\hat{v}}^{3}\in R^{n\times n}$, $R_{1,\hat{h},\hat{v}}^{4}\in R^{n\times n}$, and *T* satisfies assumption 3.

Inequality (26) can be rewritten as follows:


(27) $$\begin{array}{l} {\text{ Ξ }}_{7}+{\text{ Ξ }}_{8}<0, \end{array}$$

$$\begin{array}{l} {\text{ Ξ }}_{7}=\left[\begin{array}{cccc} \text{He(}A_{\hat{h},\hat{v}}^{*}K_{\hat{h},\hat{v}}+B^{*}F_{\hat{h},\hat{v}}^{*}\text{)} & * & * & *\\ \text{ Ξ}{\;}_{7}^{1} & -\varepsilon \text{He(}P_{\hat{h},\hat{v}}^{T}\text{)} & * & *\\ \text{ Ξ}{\;}_{7}^{2} & \varepsilon {\left(\bar{BB}\varpi_{1}\right)}^{T} & \text{He(}A_{h,v}^{*}Q_{\hat{h},\hat{v}}-\varpi_{1}\text{)} & *\\ \varepsilon_{1}{\left(\bar{BB}\varpi_{1}\right)}^{T} & \varepsilon_{1}\varepsilon {\left(\bar{BB}\varpi_{1}\right)}^{T} & Q_{\hat{h},\hat{v}}-\varepsilon_{1}\varpi_{1}^{T}-T^{*}R_{1,\hat{h},\hat{v}} & -\varepsilon_{1}\text{He}{\left(T^{*}R_{1,\hat{h},\hat{v}}\right)}^{T} \end{array}\right],\\ {\text{ Ξ }}_{8}=\text{He(}\left[\begin{array}{cccc} B^{*}\text{Δ}F_{\hat{h},\hat{v}}^{*} & 0 & 0 & 0\\ \varepsilon B^{*}\text{Δ}F_{\hat{h},\hat{v}}^{*} & 0 & 0 & 0\\ \text{Δ}A^{*}K_{\hat{h},\hat{v}}+{\left(\bar{BB}\varpi_{2}\right)}^{T} & \varepsilon {\left(\bar{BB}\varpi_{2}\right)}^{T} & \text{Δ}A^{*}Q_{\hat{h},\hat{v}}-\varpi_{2}^{T} & 0\\ \varepsilon_{1}{\left(\bar{BB}\varpi_{2}\right)}^{T} & \varepsilon_{1}\varepsilon {\left(\bar{BB}\varpi_{2}\right)}^{T} & -\varepsilon_{1}\varpi_{2}^{T} & 0 \end{array}\right]\text{)}, \end{array}$$

where $\text{ Ξ}{\;}_{7}^{1}=K_{\hat{h},\hat{v}}+\varepsilon \left(A_{\hat{h},\hat{v}}^{*}K_{\hat{h},\hat{v}}+B^{*}F_{\hat{h},\hat{v}}^{*}\right)-P_{\hat{h},\hat{v}}$, ${\text{Ξ}}_{7}^{2}=\left(A_{h,v}^{*}-A_{\hat{h},\hat{v}}^{*}\right)K_{\hat{h},\hat{v}}+{\left(\bar{BB}\varpi_{1}\right)}^{T}$, $\varpi_{1}=L_{\hat{h},\hat{v}}^{*}C^{*}T^{*}R_{1,\hat{h},\hat{v}}$, and $\varpi_{2}=\text{Δ}L_{\hat{h},\hat{v}}^{*}C^{*}T^{*}R_{1,\hat{h},\hat{v}}$.

Because $\text{Δ}L_{\hat{h},\hat{v}}^{*}=M_{L}^{*}S_{L}N_{L}^{*}L_{\hat{h},\hat{v}}^{*}$, $\text{Δ}F_{\hat{h},\hat{v}}^{*}=M_{F}S_{F}N_{F}F_{\hat{h},\hat{v}}^{*}$, and ΔA^*^ = *MF*(*t*)*N*, inequality (27) can be rewritten as:


(28) $$\begin{array}{l} {\text{Ξ}}_{7}+\text{He}\left(M_{1}S_{F}N_{1}\text{+}M_{2}F\left(t\right)N_{2}\text{+}M_{3}S_{L}N_{3}\right)<0, \end{array}$$

where $M_{1}={\left[\begin{array}{cccc} B^{*}M_{F} & \varepsilon B^{*}M_{F} & 0 & 0 \end{array}\right]}^{T}$, $N_{1}=\left[\begin{array}{cccc} N_{F}F_{\hat{h},\hat{v}}^{*} & 0 & 0 & 0 \end{array}\right]$, $M_{2}={\left[\begin{array}{cccc} 0 & 0 & M & 0 \end{array}\right]}^{T}$, $N_{2}=\left[\begin{array}{cccc} NK_{\hat{h},\hat{v}} & 0 & NQ_{\hat{h},\hat{v}} & 0 \end{array}\right]$, $N_{3}=\left[\begin{array}{cccc} 0 & 0 & N_{L}^{*}L_{\hat{h},\hat{v}}^{*}C^{*}T^{*}R_{1,\hat{h},\hat{v}} & \varepsilon_{1} \end{array}N_{L}^{*}L_{\hat{h},\hat{v}}^{*}C^{*}T^{*}R_{1,\hat{h},\hat{v}}\right]$, and *M*_3_ = ${\left[\begin{array}{cccc} \bar{BB}M_{L}^{*} & \varepsilon \bar{BB}M_{L}^{*} & -M_{L}^{*} & 0 \end{array}\right]}^{T}$.

Based on lemma 2, (28) holds if there are positive real scalars $\varepsilon_{\text{2,}\hat{h},\hat{v}}$, $\varepsilon_{3,\hat{h},\hat{v}}^{A}$, $\varepsilon_{3,\hat{h},\hat{v}}^{E}$, and $\varepsilon_{\text{4,}\hat{h},\hat{v}}$ that satisfy the following relation:


(29) $$\begin{array}{l} {\text{Ξ}}_{7}+M_{1}\varepsilon_{2,\hat{h},\hat{v}}M_{1}^{T}+N_{1}^{T}\varepsilon_{2,\hat{h},\hat{v}}^{-1}N_{1}+M_{2}\varepsilon_{3,\hat{h},\hat{v}}M_{2}^{T}+N_{2}^{T}\varepsilon_{3,\hat{h},\hat{v}}^{-1}N_{2}+M_{3}\varepsilon_{4,\hat{h},\hat{v}}M_{3}^{T}+N_{3}^{T}\varepsilon_{4,\hat{h},\hat{v}}^{-1}N_{3}<0, \end{array}$$

where $\varepsilon_{\text{3},\hat{h},\hat{v}}=\left[\begin{array}{cc} \varepsilon_{3,\hat{h},\hat{v}}^{A}I & 0\\ 0 & \varepsilon_{3,\hat{h},\hat{v}}^{E}I \end{array}\right]$. Using the Schur complement in lemma 1, (29) holds if and only if the following relation set is satisfied:


(30) $$\begin{array}{l} \left[\begin{array}{ccccccc} {\text{Ξ}}_{9}^{1} & * & * & * & * & * & *\\ {\text{Ξ}}_{9}^{2} & {\text{Ξ}}_{9}^{3} & * & * & * & * & *\\ {\text{Ξ}}_{9}^{4} & {\text{Ξ}}_{9}^{5} & {\text{Ξ}}_{9}^{6} & * & * & * & *\\ \varepsilon_{1}{\left(\bar{BB}\varpi_{1}\right)}^{T} & \varepsilon_{1}\varepsilon {\left(\bar{BB}\varpi_{1}\right)}^{T} & {\text{Ξ}}_{9}^{7} & -\varepsilon_{1}\text{He}\left(T^{*}R_{1,\hat{h},\hat{v}}\right) & * & * & *\\ N_{F}F_{\hat{h},\hat{v}}^{*} & 0 & 0 & 0 & -\varepsilon_{2,\hat{h},\hat{v}}I & * & *\\ NK_{\hat{h},\hat{v}}^{*} & 0 & NQ_{\hat{h},\hat{v}} & 0 & 0 & -\varepsilon_{3,\hat{h},\hat{v}}I & *\\ 0 & 0 & N_{L}^{*}\varpi_{1} & \varepsilon \varpi_{1} & 0 & 0 & -\varepsilon_{4,\hat{h},\hat{v}}I \end{array}\right]<0, \end{array}$$

where $\text{ Ξ}{\;}_{9}^{1}=\text{He(}A_{\hat{h},\hat{v}}^{*}K_{\hat{h},\hat{v}}+B^{*}F_{\hat{h},\hat{v}}^{*}\text{)}+B^{*}M_{F}{\;\varepsilon \;}_{2,\hat{h},\hat{v}}M_{F}^{T}B^{*T}+\bar{BB}M_{L}^{*}{\;\varepsilon \;}_{4,\hat{h},\hat{v}}M_{L}^{*T}{\bar{BB}}^{T}$, $\text{ Ξ}{\;}_{9}^{2}=\varepsilon A_{\hat{h},\hat{v}}^{*}K_{\hat{h},\hat{v}}-P_{\hat{h},\hat{v}}+$$K_{\hat{h},\hat{v}}+\varepsilon B^{*}F_{\hat{h},\hat{v}}^{*}+\varepsilon B^{*}M_{F}{\;\varepsilon \;}_{2,\hat{h},\hat{v}}M_{F}^{T}B^{*T}+\varepsilon \bar{BB}M_{L}^{*}{\;\varepsilon \;}_{4,\hat{h},\hat{v}}M_{L}^{*T}{\bar{BB}}^{T}$, $\text{ Ξ}{\;}_{9}^{3}=\varepsilon^{2}B^{*}M_{F}{\;\varepsilon \;}_{2,\hat{h},\hat{v}}M_{F}^{T}B^{*T}-\varepsilon \text{He}\left(P_{\hat{h},\hat{v}}^{T}\right)$$+\varepsilon^{2}\bar{BB}M_{L}^{*}{\;\varepsilon \;}_{4,\hat{h},\hat{v}}M_{L}^{*T}{\bar{BB}}^{T}$, ${\text{Ξ}}_{9}^{4}={\left(\bar{BB}\varpi_{1}\right)}^{T}-M_{L}^{*}\varepsilon_{4,\hat{h},\hat{v}}M_{L}^{*T}{\bar{BB}}^{T}+A_{h,v}^{*}K_{\hat{h},\hat{v}}-A_{\hat{h},\hat{v}}^{*}K_{\hat{h},\hat{v}}$, $\text{ Ξ}{\;}_{9}^{5}=\varepsilon {\left(\bar{BB}\varpi_{1}\right)}^{T}$$-\varepsilon M_{L}^{*}{\;\varepsilon \;}_{4,\hat{h},\hat{v}}M_{L}^{*T}{\bar{BB}}^{T}$, $\text{ Ξ}{\;}_{9}^{6}=\;\text{He}\left(A_{h,v}^{*}Q_{\hat{h},\hat{v}}-\varpi_{1}\right)+M{\;\varepsilon \;}_{3,\hat{h},\hat{v}}M^{T}+M_{L}^{*}{\;\varepsilon \;}_{4,\hat{h},\hat{v}}M_{L}^{*T}$, $\text{ Ξ}{\;}_{9}^{7}=Q_{\hat{h},\hat{v}}-\varepsilon_{1}\varpi_{1}^{T}-$$T^{*}R_{1,\hat{h},\hat{v}}$, and $\varpi_{1}=L_{\hat{h},\hat{v}}^{*}C^{*}T^{*}R_{1,\hat{h},\hat{v}}$.

It is assumed that $S^{*}=\left[\begin{array}{cc} S_{1}^{*} & S_{2}^{*} \end{array}\right]$; thus, the matrix $\bar{BB}$can be obtained as:


$$\begin{array}{l} \bar{BB}=\left[\begin{array}{cc} I & 0\\ -B{\left(S_{2}^{*}B\right)}^{-1}S_{1}^{*} & I-B{\left(S_{2}^{*}B\right)}^{-1}S_{2}^{*} \end{array}\right]. \end{array}$$

Then, the matrices in inequality (31) can be formulated as follows:


$$\begin{array}{c} L_{\hat{h},\hat{v}}^{*}={\left[\begin{array}{cc} 0 & L_{\hat{h},\hat{v}}^{T} \end{array}\right]}^{T},T^{*}=\left[\begin{array}{cc} T & 0\\ 0 & I \end{array}\right],\;T^{*}R_{1,\hat{h},\hat{v}}=\left[\begin{array}{cc} TR_{1,\hat{h},\hat{v}}^{1} & TR_{1,\hat{h},\hat{v}}^{2}\\ R_{1,\hat{h},\hat{v}}^{3} & R_{1,\hat{h},\hat{v}}^{4} \end{array}\right],\\ L_{\hat{h},\hat{v}}^{*}C^{*}T^{*}R_{1,\hat{h},\hat{v}}=\left[\begin{array}{cc} 0 & 0\\ L_{\hat{h},\hat{v}}CTR_{1,\hat{h},\hat{v}}^{1} & 0 \end{array}\right]=\left[\begin{array}{cc} 0_{n\times n} & 0_{n\times n}\\ \left[\begin{array}{cc} {\left(L_{\hat{h},\hat{v}}R_{1}\right)}_{n\times p} & 0_{n\times \left(n-p\right)} \end{array}\right] & 0_{n\times n} \end{array}\right],\\ \bar{BB}L_{\hat{h},\hat{v}}^{*}C^{*}T^{*}R_{1,\hat{h},\hat{v}}=\left[\begin{array}{cc} 0_{n\times n} & 0_{n\times n}\\ \left[\begin{array}{cc} {\left(\left(I-B{\left(S_{2}^{*}B\right)}^{-1}S_{2}^{*}\right)L_{\hat{h},\hat{v}}R_{1}\right)}_{n\times p} & 0_{n\times \left(n-p\right)} \end{array}\right] & 0_{n\times n} \end{array}\right]. \end{array}$$

Therefore, inequality (18) is easily obtained from the above equalities and inequality (30).

Remark 5. To avoid introducing the derivative of the membership function in the derivative of the Lyapunov function, *P*_1_ is chosen as a constant matrix. The $P_{3,\hat{h},\hat{v}}$ and $P_{\text{4,}\hat{h},\hat{v}}$ in $P_{\hat{h},\hat{v}}$ are related to the membership functions $v_{k}\left(\hat{\xi }\left(t\right)\right)$ and *h*_*i*_(ẑ(*t*)). Consequently, the conservativeness of this approach is significantly reduced. Moreover, the Lyapunov matrices and the observer gain matrices are decoupled by Finsler's lemma.

T-S fuzzy descriptor systems may occasionally have different output matrices, so the following theorem gives the relaxed sufficient LMI conditions for T-S fuzzy descriptor systems with different output matrices to expand the application scope of the design theory given in this study.

Theorem 2: Suppose that the T-S fuzzy descriptor system matrices in (2) satisfy ΔA^*^ ≠ 0 and *C*_*h*_ ≠ *C*_$\hat{h}$_. Given constants ε > 0, ε_1_ > 0 and ε_2_ > 0, the closed-loop systems (14, 15) are asymptotically stable if positive definite matrices *P*_1_ and *Q*_1_, scalars ε_3,*i,k*_, $\varepsilon_{\text{4},i,k}^{E}$, $\varepsilon_{\text{4,}i,k}^{A}$, and ε_5,*i,k*_, and a set of matrices *P*_3,*i,k*_, *P*_4,*i,k*_, *F*_*i,k*_, *K*_1,*k*_, *K*_2,*k*_, *K*_3,*i*_, *K*_4,*i*_, *L*_*i,k*_, *R*_1_, $R_{1,i,k}^{11}$, $R_{1,i,k}^{12}$, $R_{2,i,k}^{11}$, $R_{2,i,k}^{12}$,$R_{1,i,k}^{2}$, $R_{1,i,k}^{3}$, $R_{1,i,k}^{4}$, $R_{2,i,k}^{2}$, $R_{2,i,k}^{3}$, and $R_{2,i,k}^{4}$exist, where *k* ∈ {1, ⋯ , *r*_*e*_} and *i* ∈ {1, ⋯ , *r*}, such that the following LMIs hold:


(31) $$\begin{array}{l} \left[\begin{array}{cc} {\text{Ω}}_{1} & *\\ {\text{Ω}}_{2} & {\text{Ω}}_{3} \end{array}\right]<0, \end{array}$$

$$\begin{array}{l} {\text{Ω}}_{1}=\left[\begin{array}{cccccccccc} {\text{Δ}}_{1,1} & * & * & * & * & * & * & * & * & *\\ {\text{Δ}}_{2,1} & {\text{Δ}}_{2,2} & * & * & * & * & * & * & * & *\\ {\text{Δ}}_{3,1} & {\text{Δ}}_{3,2} & {\text{Δ}}_{3,3} & * & * & * & * & * & * & *\\ {\text{Δ}}_{4,1} & {\text{Δ}}_{4,2} & {\text{Δ}}_{4,3} & {\text{Δ}}_{4,4} & * & * & * & * & * & *\\ 0 & {\text{Δ}}_{5,2} & 0 & {\text{Δ}}_{5,4} & {\text{Δ}}_{5,5} & * & * & * & * & *\\ {\text{Δ}}_{6,1} & {\text{Δ}}_{6,2} & -Y_{\hat{h},\hat{v}} & {\text{Δ}}_{6,4} & {\text{Δ}}_{6,5} & {\text{Δ}}_{6,6} & * & * & * & *\\ K_{1,\hat{v}} & {\text{Δ}}_{7,2} & 0 & {\text{Δ}}_{7,4} & {\text{Δ}}_{7,5} & {\text{Δ}}_{7,6} & {\text{Δ}}_{7,7} & * & * & *\\ K_{3,\hat{h}} & K_{4,\hat{h}} & 0 & 0 & {\text{Δ}}_{8,5} & {\text{Δ}}_{8,6} & {\text{Δ}}_{8,7} & {\text{Δ}}_{8,8} & * & *\\ -K_{1,\hat{v}} & {\text{Δ}}_{92} & -T_{\hat{h}}R_{2,\hat{h},\hat{v}}^{1} & {\text{Δ}}_{9,4} & 0 & {\text{Δ}}_{9,6} & 0 & 0 & {\text{Δ}}_{9,9} & *\\ -K_{3,\hat{h}} & K_{4,\hat{h}} & -R_{2,\hat{h},\hat{v}}^{3} & -R_{2,\hat{h},\hat{v}}^{4} & 0 & 0 & 0 & 0 & {\text{Δ}}_{10,9} & -\varepsilon_{2}\text{He(}T_{\hat{h}}R_{2,\hat{h},\hat{v}}^{4}\text{)} \end{array}\right],\\ {\text{ Ω }}_{2}=\left[\begin{array}{cccccccccc} N_{F}F_{\hat{h},\hat{v}} & 0 & 0 & 0 & 0 & 0 & 0 & 0 & 0 & 0\\ N_{A}K_{1,\hat{v}} & N_{A}K_{2,\hat{v}} & 0 & 0 & N_{A} & 0 & 0 & 0 & 0 & 0\\ N_{E}K_{3,\hat{h}} & N_{E}K_{4,\hat{h}} & 0 & 0 & N_{E}Q_{3,\hat{h}} & N_{E}Q_{4,\hat{h}} & 0 & 0 & 0 & 0\\ 0 & 0 & N_{L}Y_{\hat{h},\hat{v}} & 0 & N_{L}Y_{\hat{h},\hat{v}} & 0 & \varepsilon_{1}N_{L}Y_{\hat{h},\hat{v}} & 0 & \varepsilon_{2}N_{L}Y_{\hat{h},\hat{v}} & 0 \end{array}\right],\\ {\text{ Ω }}_{3}=\left[\begin{array}{cccc} -{\;\varepsilon \;}_{3,\hat{h},\hat{v}}I & * & * & *\\ 0 & -\;\varepsilon {\;}_{4,\hat{h},\hat{v}}^{A}I & * & *\\ 0 & 0 & -\;\varepsilon {\;}_{4,\hat{h},\hat{v}}^{E}I & *\\ 0 & 0 & 0 & -{\;\varepsilon \;}_{5,\hat{h},\hat{v}}I \end{array}\right], \end{array}$$

where Δ_1,1_ = He(*K*_3,$\hat{h}$_), ${\text{Δ}}_{2,1}=A_{\hat{h}}K_{1,\hat{v}}-E_{\hat{v}}K_{3,\hat{h}}+BF_{\hat{h},\hat{v}}+K_{4,\hat{h}}^{T}$, ${\text{Δ}}_{2,2}=BM_{F}{\;\varepsilon \;}_{3,\hat{h},\hat{v}}M_{F}^{T}B^{T}+\text{He(}A_{\hat{h}}K_{2,\hat{v}})-$$\text{He}(E_{\hat{v}}K_{4,\hat{h}}\text{)}+\bar{B}M_{L}{\;\varepsilon \;}_{5,\hat{h},\hat{v}}M_{L}^{T}{\bar{B}}^{T}$, ${\text{Δ}}_{3,1}=K_{1,\hat{v}}+\varepsilon K_{3,\hat{h}}-P_{1}$, ${\text{Δ}}_{3,2}=K_{2,\hat{v}}+\varepsilon K_{4,\hat{h}}$, Δ_3,3_ = −εHe(*P*_1_), ${\text{Δ}}_{4,1}=K_{3,\hat{h}}+\varepsilon \left(A_{\hat{h}}K_{1,\hat{v}}-E_{\hat{v}}K_{3,\hat{h}}+BF_{\hat{h},\hat{v}}\right)-P_{3,\hat{h},\hat{v}}$,${\text{Δ}}_{4,2}=K_{4,\hat{h}}+\varepsilon \left(A_{\hat{h}}K_{2,\hat{v}}-E_{\hat{v}}K_{4,\hat{h}}\right)+\varepsilon BM_{F}{\;\varepsilon \;}_{3,\hat{h},\hat{v}}M_{F}^{T}B^{T}-$$P_{4,\hat{h},\hat{v}}+\varepsilon \bar{B}M_{L}{\;\varepsilon \;}_{5,\hat{h},\hat{v}}M_{L}^{T}{\bar{B}}^{T}$, ${\text{Δ}}_{4,3}=-\varepsilon P_{3,\hat{h},\hat{v}}+\varepsilon \bar{B}Y_{\hat{h},\hat{v}}$, ${\text{Δ}}_{4,4}=\varepsilon^{2}BM_{F}{\;\varepsilon \;}_{3,\hat{h},\hat{v}}M_{F}^{T}B^{T}+\varepsilon^{2}\bar{B}M_{L}{\;\varepsilon \;}_{5,\hat{h},\hat{v}}M_{L}^{T}{\bar{B}}^{T}-$ $\varepsilon \text{He(}P_{4,\hat{h},\hat{v}}\text{)}$, ${\text{Δ}}_{5,2}={\left(\bar{B}Y_{\hat{h},\hat{v}}\right)}^{T}$, ${\text{Δ}}_{5,4}=\varepsilon {\left(\bar{B}Y_{\hat{h},\hat{v}}\right)}^{T}$, Δ_5,5_ = He(*Q*_3,$\hat{h}$_), ${\text{Δ}}_{6,1}=A_{h}K_{1,\hat{v}}-A_{\hat{h}}K_{1,\hat{v}}-$$\left(E_{v}-E_{\hat{v}}\right)K_{3,\hat{h}}$, ${\text{Δ}}_{6,2}=-\left(E_{v}-E_{\hat{v}}\right)K_{4,\hat{h}}-M_{L}{\;\varepsilon \;}_{5,\hat{h},\hat{v}}M_{L}^{T}{\bar{B}}^{T}+\left(A_{h}-A_{\hat{h}}\right)K_{2,\hat{v}}$, ${\text{Δ}}_{6,4}=-\varepsilon M_{L}{\;\varepsilon \;}_{5,\hat{h},\hat{v}}M_{L}^{T}{\bar{B}}^{T}$, ${\text{Δ}}_{6,5}=A_{h}Q_{1}-E_{v}Q_{3,\hat{h}}+Q_{4,\hat{h}}^{T}-Y_{\hat{h},\hat{v}}$, ${\text{Δ}}_{6,6}=-\text{He(}E_{v}Q_{4,\hat{h}}\text{)}+M_{L}{\;\varepsilon \;}_{5,\hat{h},\hat{v}}M_{L}^{T}+M_{A}\;\varepsilon {\;}_{4,\hat{h},\hat{v}}^{A}M_{A}^{T}+M_{E}\;\varepsilon {\;}_{4,\hat{h},\hat{v}}^{E}M_{E}^{T}$,${\text{Δ}}_{7,2}=K_{2,\hat{v}}+\varepsilon_{1}{\left(\bar{B}Y_{\hat{h},\hat{v}}\right)}^{T}$, ${\text{Δ}}_{7,4}=\varepsilon_{1}\varepsilon {\left(\bar{B}Y_{\hat{h},\hat{v}}\right)}^{T}$, ${\text{Δ}}_{7,5}=Q_{1}-TR_{1,\hat{h},\hat{v}}^{1}$, ${\text{Δ}}_{7,6}=-\varepsilon_{1}{\left(Y_{\hat{h},\hat{v}}\right)}^{T}-TR_{1,\hat{h},\hat{v}}^{2}$, Δ_7,7_=$-\varepsilon_{1}\text{He(}TR_{1,\hat{h},\hat{v}}^{1}\text{)}$, ${\text{Δ}}_{8,5}=Q_{3,\hat{h}}-R_{1,\hat{h},\hat{v}}^{3}$, ${\text{Δ}}_{8,6}=Q_{4,\hat{h}}-R_{1,\hat{h},\hat{v}}^{4}$, ${\text{Δ}}_{8,7}=-\varepsilon_{1}R_{1,\hat{h},\hat{v}}^{3}-\varepsilon_{1}{\left(TR_{1,\hat{h},\hat{v}}^{2}\right)}^{T}$, Δ_8,8_=$-\varepsilon_{1}\text{He(}R_{1,\hat{h},\hat{v}}^{4}\text{)}$, ${\text{Δ}}_{9,2}=-K_{2,\hat{v}}+\varepsilon_{2}{\left(\bar{B}Y_{\hat{h},\hat{v}}\right)}^{T}$, ${\text{Δ}}_{9,4}=-T_{\hat{h}}R_{2,\hat{h},\hat{v}}^{2}+\varepsilon_{2}\varepsilon {\left(\bar{B}Y_{\hat{h},\hat{v}}\right)}^{T}$, ${\text{Δ}}_{9,6}=-\varepsilon_{2}{\left(Y_{\hat{h},\hat{v}}\right)}^{T}$, Δ_9,9_=$-\varepsilon_{2}\text{He(}T_{\hat{h}}R_{2,\hat{h},\hat{v}}^{1}\text{)}$, ${\text{Δ}}_{10,9}=-\varepsilon_{2}R_{2,\hat{h},\hat{v}}^{3}-\varepsilon_{2}{\left(T_{\hat{h}}R_{2,\hat{h},\hat{v}}^{2}\right)}^{T}$, $Y_{\hat{h},\hat{v}}=\left[\begin{array}{cc} {\left(L_{\hat{h},\hat{v}}R_{1}\right)}_{n\times p} & 0_{n\times \left(n-p\right)} \end{array}\right]$,$\bar{B}=-B{\left(S_{2}^{*}B\right)}^{-1}S_{2}^{*}$+*I*, $R_{1,\hat{h},\hat{v}}^{1}=\left[\begin{array}{cc} R_{1} & 0_{p\times \left(n-p\right)}\\ R_{1,\hat{h},\hat{v}}^{11} & R_{1,\hat{h},\hat{v}}^{12} \end{array}\right]$, $R_{2,\hat{h},\hat{v}}^{1}=\left[\begin{array}{cc} R_{1} & 0_{p\times \left(n-p\right)}\\ R_{2,\hat{h},\hat{v}}^{11} & R_{2,\hat{h},\hat{v}}^{12} \end{array}\right]$, $R_{1,\hat{h},\hat{v}}^{2}=\left[\begin{array}{c} 0_{p\times n}\\ R_{1,\hat{h},\hat{v}}^{21} \end{array}\right]$, and $R_{2,\hat{h},\hat{v}}^{2}=\left[\begin{array}{c} 0_{p\times n}\\ R_{2,\hat{h},\hat{v}}^{21} \end{array}\right]$.

Proof: Under the conditions of *C*_*h*_ ≠ *C*_$\hat{h}$_, the constructed Lyapunov function candidate is the same as in Equation (19). However, the sliding mode dynamics (Equations 14 and 15) under the condition of *C*_*h*_ ≠ *C*_$\hat{h}$_are different from the sliding mode dynamics (Equations 12 and 13) under the conditions of $C_{h}=C_{\hat{h}}=C^{*}$. The derivative of the Lyapunov function is calculated based on the sliding mode dynamics. Therefore, the derivative of the Lyapunov function candidate (19) under the condition of *C*_*h*_ ≠ *C*_$\hat{h}$_can be obtained as follows:


(32) $$\begin{array}{l} \overset{∙}{V}\left(\hat{x}\left(t\right),e_{1}\left(t\right)\right)={\overset{∙}{\hat{X}}}^{*T}\left(t\right)E^{*T}P_{\hat{h},\hat{v}}^{-1}{\hat{X}}^{*}\left(t\right)+{\hat{X}}^{*T}\left(t\right)P_{\hat{h},\hat{v}}^{-T}E^{*}{\overset{∙}{\hat{X}}}^{*}\left(t\right)+{\dot{e}}^{T}\left(t\right)E^{*T}Q_{\hat{h},\hat{v}}^{-1}e\left(t\right)+e^{T}\left(t\right)Q_{\hat{h},\hat{v}}^{-T}E^{*}\dot{e}\left(t\right) \end{array}$$

$$\begin{array}{c} ={\left[\begin{array}{c} {\hat{X}}^{*}\left(t\right)\\ E^{*}{\overset{∙}{\hat{X}}}^{*}\left(t\right)\\ e\left(t\right) \end{array}\right]}^{T}{\text{Ψ}}_{1}\left[\begin{array}{c} {\hat{X}}^{*}\left(t\right)\\ E^{*}{\overset{∙}{\hat{X}}}^{*}\left(t\right)\\ e\left(t\right) \end{array}\right]<0.\\ {\text{Ψ}}_{1}=\left[\begin{array}{ccc} 0 & * & *\\ P_{\hat{h}\hat{v}}^{-1} & 0 & *\\ Q_{\hat{h},\hat{v}}^{-T}\left(A_{h,v}^{*}-A_{\hat{h},\hat{v}}^{*}+\text{Δ}A^{*}-\left(L_{\hat{h},\hat{v}}^{*}+\text{Δ}L_{\hat{h},\hat{v}}^{*}\right)\left(C_{h}^{*}-C_{\hat{h}}^{*}\right)\right) & 0 & \text{He}\left(Q_{\hat{h},\hat{v}}^{-T}\left(A_{h,v}^{*}-\left(L_{\hat{h},\hat{v}}^{*}+\text{Δ}L_{\hat{h},\hat{v}}^{*}\right)C_{h}^{*}\right)\right) \end{array}\right] \end{array}$$

If (32) holds, then the following inequality can be obtained by Finsler's lemma (lemma 3) under the constraint in Equation (17).


(33) $$\begin{array}{l} {\text{Ψ}}_{1}+\text{He(}\left[\begin{array}{c} U_{\hat{h},\hat{v}}\\ V_{\hat{h},\hat{v}}\\ W_{\hat{h},\hat{v}} \end{array}\right]\left[\begin{array}{ccc} A_{\hat{h},\hat{v}}^{*}+B^{*}\left(F_{\hat{h},\hat{v}}^{*}+\text{Δ}F_{\hat{h},\hat{v}}^{*}\right)K_{\hat{h},\hat{v}}^{-1}+\bar{BB}\left(L_{\hat{h},\hat{v}}^{*}+\text{Δ}L_{\hat{h},\hat{v}}^{*}\right)\left(C_{h}^{*}-C_{\hat{h}}^{*}\right) & -I & \bar{BB}\left(L_{\hat{h},\hat{v}}^{*}+\text{Δ}L_{\hat{h},\hat{v}}^{*}\right)C_{{\;}^{h}}^{*} \end{array}\right]\text{)}<0, \end{array}$$

where $U_{\hat{h},\hat{v}}$, $V_{\hat{h},\hat{v}}$, and $W_{\hat{h},\hat{v}}$ are matrix variables with appropriate dimensions. By pre- and post-multiplying (33) by $\left[\begin{array}{ccc} K_{\hat{h},\hat{v}}^{T} & 0 & 0\\ 0 & P_{\hat{h},\hat{v}} & 0\\ 0 & 0 & Q_{\hat{h},\hat{v}}^{T} \end{array}\right]$and $\left[\begin{array}{ccc} K_{\hat{h},\hat{v}} & 0 & 0\\ 0 & P_{\hat{h},\hat{v}}^{T} & 0\\ 0 & 0 & Q_{\hat{h},\hat{v}} \end{array}\right]$, respectively, the following inequality is obtained:


(34) $$\begin{array}{l} {\text{Ψ}}_{2}+{\text{Ψ}}_{3}<0, \end{array}$$

$$\begin{array}{c} {\text{Ψ}}_{2}=\left[\begin{array}{ccc} 0 & * & *\\ K_{\hat{h},\hat{v}} & 0 & *\\ \left(A_{h,v}^{*}-A_{\hat{h},\hat{v}}^{*}+\text{Δ}A^{*}-\left(L_{\hat{h},\hat{v}}^{*}+\text{Δ}L_{\hat{h},\hat{v}}^{*}\right)\left(C_{h}^{*}-C_{\hat{h}}^{*}\right)\right)K_{\hat{h},\hat{v}} & 0 & \text{He(}\left(A_{h,v}^{*}-\left(L_{\hat{h},\hat{v}}^{*}+\text{Δ}L_{\hat{h},\hat{v}}^{*}\right)C_{h}^{*}\right)Q_{\hat{h},\hat{v}}+\text{Δ}A^{*}) \end{array}\right],\\ {\text{Ψ}}_{3}\text{=He(}\left[\begin{array}{c} K_{\hat{h},\hat{v}}^{T}U_{\hat{h},\hat{v}}\\ P_{\hat{h},\hat{v}}V_{\hat{h},\hat{v}}\\ Q_{\hat{h},\hat{v}}^{T}W_{\hat{h},\hat{v}} \end{array}\right]\left[\begin{array}{ccc} {\text{Ψ}}_{3}^{1} & -P_{\hat{h},\hat{v}}^{T} & \bar{BB}\left(L_{\hat{h},\hat{v}}^{*}+\text{Δ}L_{\hat{h},\hat{v}}^{*}\right)C_{h}^{*}Q_{\hat{h},\hat{v}} \end{array}\right]\text{)}, \end{array}$$

where ${\text{Ψ}}_{3}^{1}\text{=}A_{\hat{h},\hat{v}}^{*}K_{\hat{h},\hat{v}}+B^{*}\left(F_{\hat{h},\hat{v}}^{*}+\text{Δ}F_{\hat{h},\hat{v}}^{*}\right)+\bar{BB}\left(L_{\hat{h},\hat{v}}^{*}+\text{Δ}L_{\hat{h},\hat{v}}^{*}\right)\left(C_{h}^{*}-C_{\hat{h}}^{*}\right)K_{\hat{h},\hat{v}}$.

Define $U_{\hat{h},\hat{v}}=K_{\hat{h},\hat{v}}^{-T}$, $V_{\hat{h},\hat{v}}=\varepsilon P_{\hat{h},\hat{v}}^{-1}$, $W_{\hat{h},\hat{v}}=0$, and ε > 0. Then, inequality (34) can be represented as:


(35) $$\begin{array}{l} {\text{Ψ}}_{4}+{\text{Ψ}}_{5}<0, \end{array}$$

$$\begin{array}{c} {\text{Ψ}}_{4}=\left[\begin{array}{ccc} \text{He(}A_{\hat{h},\hat{v}}^{*}K_{\hat{h},\hat{v}}+B^{*}\left(F_{\hat{h},\hat{v}}^{*}+\text{Δ}F_{\hat{h},\hat{v}}^{*}\right)\text{)} & * & *\\ K_{\hat{h},\hat{v}}+\varepsilon \left(A_{\hat{h},\hat{v}}^{*}K_{\hat{h},\hat{v}}+B^{*}\left(F_{\hat{h},\hat{v}}^{*}+\text{Δ}F_{\hat{h},\hat{v}}^{*}\right)\right)-P_{\hat{h},\hat{v}} & -\varepsilon \text{He(}P_{\hat{h},\hat{v}}\text{)} & *\\ \left(A_{h,v}^{*}-A_{\hat{h},\hat{v}}^{*}\right)K_{\hat{h},\hat{v}}+\text{Δ}A^{*}K_{\hat{h},\hat{v}} & 0 & \text{He}\left(\text{Δ}A^{*}Q_{\hat{h},\hat{v}}+A_{h,v}^{*}Q_{\hat{h},\hat{v}}\right) \end{array}\right],\\ {\text{Ψ}}_{5}=\left[\begin{array}{ccc} \text{He}\left(\bar{BB}\left(L_{\hat{h},\hat{v}}^{*}+\text{Δ}L_{\hat{h},\hat{v}}^{*}\right)\left(C_{h}^{*}-C_{\hat{h}}^{*}\right)K_{\hat{h},\hat{v}}\right) & * & *\\ \varepsilon \bar{BB}\left(L_{\hat{h},\hat{v}}^{*}+\text{Δ}L_{\hat{h},\hat{v}}^{*}\right)\left(C_{h}^{*}-C_{\hat{h}}^{*}\right)K_{\hat{h},\hat{v}} & 0 & *\\ {\text{Ψ}}_{5}^{1} & \varepsilon {\left(\bar{BB}\left(L_{\hat{h},\hat{v}}^{*}+\text{Δ}L_{\hat{h},\hat{v}}^{*}\right)C_{h}^{*}Q_{\hat{h},\hat{v}}\right)}^{T} & -\text{He}{\left(\left(L_{\hat{h},\hat{v}}^{*}+\text{Δ}L_{\hat{h},\hat{v}}^{*}\right)C_{h}^{*}Q_{\hat{h},\hat{v}}\right)}^{T} \end{array}\right], \end{array}$$

where ${\text{Ψ}}_{5}^{1}={\left(\bar{BB}\left(L_{\hat{h},\hat{v}}^{*}+\text{Δ}L_{\hat{h},\hat{v}}^{*}\right)C_{h}^{*}Q_{\hat{h},\hat{v}}\right)}^{T}-\left(L_{\hat{h},\hat{v}}^{*}+\text{Δ}L_{\hat{h},\hat{v}}^{*}\right)\left(C_{h}^{*}-C_{\hat{h}}^{*}\right)K_{\hat{h},\hat{v}}$.

The corresponding auxiliary variables are introduced into the system, and the dimension of the system is increased with the augmented method to eliminate the coupling between the Lyapunov function matrix $Q_{\hat{h},\hat{v}}$ and the observer gain matrix $L_{\hat{h},\hat{v}}^{*}$ in inequality (35). The following equations are defined:


(36) $$\begin{array}{l} x_{4}\left(t\right)={\left(\bar{BB}\left(L_{\hat{h},\hat{v}}^{*}+\text{Δ}L_{\hat{h},\hat{v}}^{*}\right)C_{h}^{*}\right)}^{T}x_{1}\left(t\right)+\varepsilon {\left(\bar{BB}\left(L_{\hat{h},\hat{v}}^{*}+\text{Δ}L_{\hat{h},\hat{v}}^{*}\right)C_{h}^{*}\right)}^{T}x_{2}\left(t\right)-{\left(\left(L_{\hat{h},\hat{v}}^{*}+\text{Δ}L_{\hat{h},\hat{v}}^{*}\right)C_{h}^{*}\right)}^{T}x_{3}\left(t\right), \end{array}$$

(37) $$\begin{array}{l} x_{5}\left(t\right)={\left(\bar{BB}\left(L_{\hat{h},\hat{v}}^{*}+\text{Δ}L_{\hat{h},\hat{v}}^{*}\right)C_{\hat{h}}^{*}\right)}^{T}x_{1}\left(t\right)+\varepsilon {\left(\bar{BB}\left(L_{\hat{h},\hat{v}}^{*}+\text{Δ}L_{\hat{h},\hat{v}}^{*}\right)C_{\hat{h}}^{*}\right)}^{T}x_{2}\left(t\right)-{\left(\left(L_{\hat{h},\hat{v}}^{*}+\text{Δ}L_{\hat{h},\hat{v}}^{*}\right)C_{\hat{h}}^{*}\right)}^{T}x_{3}\left(t\right). \end{array}$$

Thus, inequality (35) can be expressed as:


(38) $$\begin{array}{l} {\left[\begin{array}{c} x_{1}\left(t\right)\\ x_{2}\left(t\right)\\ x_{3}\left(t\right)\\ x_{4}\left(t\right)\\ x_{5}\left(t\right) \end{array}\right]}^{T}{\text{Ψ}}_{6}\left[\begin{array}{c} x_{1}\left(t\right)\\ x_{2}\left(t\right)\\ x_{3}\left(t\right)\\ x_{4}\left(t\right)\\ x_{5}\left(t\right) \end{array}\right]<0, \end{array}$$

$$\begin{array}{l} {\text{Ψ}}_{6}=\left[\begin{array}{ccccc} \text{He(}A_{\hat{h},\hat{v}}^{*}K_{\hat{h},\hat{v}}+B^{*}\left(F_{\hat{h},\hat{v}}^{*}+\text{Δ}F_{\hat{h},\hat{v}}^{*}\right)\text{)} & * & * & * & *\\ K_{\hat{h},\hat{v}}+\varepsilon \left(A_{\hat{h},\hat{v}}^{*}K_{\hat{h},\hat{v}}+B^{*}\left(F_{\hat{h},\hat{v}}^{*}+\text{Δ}F_{\hat{h},\hat{v}}^{*}\right)\right)-P_{\hat{h},\hat{v}} & -\varepsilon \text{He(}P_{\hat{h},\hat{v}}\text{)} & * & * & *\\ \left(A_{h,v}^{*}-A_{\hat{h},\hat{v}}^{*}\right)K_{\hat{h},\hat{v}}+\text{Δ}A^{*}K_{\hat{h},\hat{v}} & 0 & \text{He(Δ}A^{*}Q_{\hat{h},\hat{v}}+A_{h,v}^{*}Q_{\hat{h},\hat{v}}) & * & *\\ K_{\hat{h},\hat{v}} & 0 & Q_{\hat{h},\hat{v}} & 0 & *\\ -K_{\hat{h},\hat{v}} & 0 & 0 & 0 & 0 \end{array}\right] \end{array}$$

If (38) holds, then the following inequality can be obtained by Finsler's lemma (lemma 3) under the constraints in Equations (36, 37).


(39) $$\begin{array}{l} {\text{Ψ}}_{7}+\text{He}\left({\text{Ψ}}_{8}\right)<0, \end{array}$$

$$\begin{array}{l} {\text{Ψ}}_{7}=\left[\begin{array}{ccccc} \text{He(}A_{\hat{h},\hat{v}}^{*}K_{\hat{h},\hat{v}}+B^{*}\left(F_{\hat{h},\hat{v}}^{*}+\text{Δ}F_{\hat{h},\hat{v}}^{*}\right)\text{)} & * & * & * & *\\ K_{\hat{h},\hat{v}}-P_{\hat{h},\hat{v}}+\varepsilon \left(A_{\hat{h},\hat{v}}^{*}K_{\hat{h},\hat{v}}+B^{*}\left(F_{\hat{h},\hat{v}}^{*}+\text{Δ}F_{\hat{h},\hat{v}}^{*}\right)\right) & -\varepsilon \text{He(}P_{\hat{h},\hat{v}}\text{)} & * & * & *\\ \left(A_{h,v}^{*}-A_{\hat{h},\hat{v}}^{*}\right)K_{\hat{h},\hat{v}}+\text{Δ}A^{*}K_{\hat{h},\hat{v}} & 0 & \text{He}\left(A_{h,v}^{*}Q_{\hat{h},\hat{v}}+\text{Δ}A^{*}Q_{\hat{h},\hat{v}}\right) & * & *\\ K_{\hat{h},\hat{v}} & 0 & Q_{\hat{h},\hat{v}} & 0 & *\\ -K_{\hat{h},\hat{v}} & 0 & 0 & 0 & 0 \end{array}\right],\\ {\text{Ψ}}_{8}=\left[\begin{array}{cc} 0 & 0\\ 0 & {\left(T_{\hat{h}}^{*}R_{2,\hat{h},\hat{v}}^{*}\right)}^{T}\\ {\left(T_{h}^{*}R_{1,\hat{h},\hat{v}}^{*}\right)}^{T} & 0\\ \varepsilon_{1}{\left(T_{h}^{*}R_{1,\hat{h},\hat{v}}^{*}\right)}^{T} & 0\\ 0 & \varepsilon_{2}{\left(T_{\hat{h}}^{*}R_{2,\hat{h},\hat{v}}^{*}\right)}^{T} \end{array}\right]\left[\begin{array}{ccccc} {\text{Ψ}}_{8}^{1} & \varepsilon {\text{Ψ}}_{8}^{1} & -{\left(\left(L_{\hat{h},\hat{v}}^{*}+\text{Δ}L_{\hat{h},\hat{v}}^{*}\right)C_{h}^{*}\right)}^{T} & -I & 0\\ {\text{Ψ}}_{8}^{2} & \varepsilon {\text{Ψ}}_{8}^{2} & -{\left(\left(L_{\hat{h},\hat{v}}^{*}+\text{Δ}L_{\hat{h},\hat{v}}^{*}\right)C_{\hat{h}}^{*}\right)}^{T} & 0 & -I \end{array}\right], \end{array}$$

where ${\text{Ψ}}_{8}^{1}={\left(\bar{BB}\left(L_{\hat{h},\hat{v}}^{*}+\text{Δ}L_{\hat{h},\hat{v}}^{*}\right)C_{h}^{*}\right)}^{T}$,${\text{Ψ}}_{8}^{2}={\left(\bar{BB}\left(L_{\hat{h},\hat{v}}^{*}+\text{Δ}L_{\hat{h},\hat{v}}^{*}\right)C_{\hat{h}}^{*}\right)}^{T}$, $R_{1,\hat{h},\hat{v}}=\left[\begin{array}{cc} R_{1,\hat{h},\hat{v}}^{1} & R_{1,\hat{h},\hat{v}}^{2}\\ R_{1,\hat{h},\hat{v}}^{3} & R_{1,\hat{h},\hat{v}}^{4} \end{array}\right]$, $R_{2,\hat{h},\hat{v}}=\left[\begin{array}{cc} R_{2,\hat{h},\hat{v}}^{1} & R_{2,\hat{h},\hat{v}}^{2}\\ R_{2,\hat{h},\hat{v}}^{3} & R_{2,\hat{h},\hat{v}}^{4} \end{array}\right]$, $R_{1,\hat{h},\hat{v}}^{1}=\left[\begin{array}{cc} R_{1} & 0_{p\times \left(n-p\right)}\\ R_{1,\hat{h},\hat{v}}^{11} & R_{1,\hat{h},\hat{v}}^{12} \end{array}\right]$, $R_{2,\hat{h},\hat{v}}^{1}=\left[\begin{array}{cc} R_{1} & 0_{p\times \left(n-p\right)}\\ R_{2,\hat{h},\hat{v}}^{11} & R_{2,\hat{h},\hat{v}}^{12} \end{array}\right]$, $T_{h}^{*}=\left[\begin{array}{cc} T_{h} & 0\\ 0 & I \end{array}\right]$, $T_{\overset{⌢}{h}}^{*}=\left[\begin{array}{cc} T_{\overset{⌢}{h}} & 0\\ 0 & I \end{array}\right]$, $R_{1}\in R^{p\times p}$, $R_{1,\hat{h},\hat{v}}^{11}\in R^{\left(n-p\right)\times p}$, $R_{1,\hat{h},\hat{v}}^{12}\in R^{\left(n-p\right)\times \left(n-p\right)}$, $R_{2,\hat{h},\hat{v}}^{11}\in R^{\left(n-p\right)\times p}$, $R_{2,\hat{h},\hat{v}}^{12}\in R^{\left(n-p\right)\times \left(n-p\right)}$, $R_{1,\hat{h},\hat{v}}^{2}\in R^{n\times n}$, $R_{1,\hat{h},\hat{v}}^{3}\in R^{n\times n}$, $R_{1,\hat{h},\hat{v}}^{4}\in R^{n\times n}$, $R_{2,\hat{h},\hat{v}}^{2}\in R^{n\times n}$, $R_{2,\hat{h},\hat{v}}^{3}\in R^{n\times n}$, $R_{2,\hat{h},\hat{v}}^{4}\in R^{n\times n}$, and *T*_*h*_ and *T*_$\hat{h}$_ satisfy assumption 3.

Inequality (39) can be rewritten as follows:


(40) $$\begin{array}{l} {\text{Ψ}}_{9}+\text{He}\left(\left[\begin{array}{ccccc} B^{*}\text{Δ}F_{\hat{h},\hat{v}}^{*} & 0 & 0 & 0 & 0\\ \varepsilon B^{*}\text{Δ}F_{\hat{h},\hat{v}}^{*}+{\left(\bar{BB}\varpi_{4}\right)}^{T} & \varepsilon {\left(\bar{BB}\varpi_{4}\right)}^{T} & -{\varpi_{4}}^{T} & 0 & 0\\ \text{Δ}A^{*}K_{\hat{h},\hat{v}}+{\left(\bar{BB}\varpi_{3}\right)}^{T} & \varepsilon {\left(\bar{BB}\varpi_{3}\right)}^{T} & \text{Δ}A^{*}Q_{\hat{h},\hat{v}}-{\varpi_{3}}^{T} & 0 & 0\\ \varepsilon_{1}{\left(\bar{BB}\varpi_{3}\right)}^{T} & \varepsilon_{1}\varepsilon {\left(\bar{BB}\varpi_{3}\right)}^{T} & -\varepsilon_{1}{\varpi_{3}}^{T} & 0 & 0\\ \varepsilon_{2}{\left(\bar{BB}\varpi_{4}\right)}^{T} & \varepsilon_{2}\varepsilon {\left(\bar{BB}\varpi_{4}\right)}^{T} & -\varepsilon_{2}{\varpi_{4}}^{T} & 0 & 0 \end{array}\right]\right)<0, \end{array}$$

$$\begin{array}{l} {\text{Ψ}}_{9}=\left[\begin{array}{ccccc} {\text{Ψ}}_{9}^{1} & * & * & * & *\\ {\text{Ψ}}_{9}^{2} & {\text{Ψ}}_{9}^{3} & * & * & *\\ {\text{Ψ}}_{9}^{4} & {\text{Ψ}}_{9}^{5} & \text{He(}A_{h,v}^{*}Q_{\hat{h},\hat{v}}-\varpi_{1}\text{)} & * & *\\ {\text{Ψ}}_{9}^{6} & {\text{Ψ}}_{9}^{7} & -T_{h}^{*}R_{1,\hat{h},\hat{v}}+Q_{\hat{h},\hat{v}}-\varepsilon_{1}\varpi_{1}^{T} & -\varepsilon_{1}\text{He}{\left(T_{h}^{*}R_{1,\hat{h},\hat{v}}\right)}^{T} & *\\ {\text{Ψ}}_{9}^{8} & {\text{Ψ}}_{9}^{9} & -\varepsilon_{2}\varpi_{2}^{T} & 0 & -\varepsilon_{2}\text{He}{\left(T_{\hat{h}}^{*}R_{2,\hat{h},\hat{v}}\right)}^{T} \end{array}\right], \end{array}$$

where ${\text{Ψ}}_{9}^{1}=\text{He(}A_{\hat{h},\hat{v}}^{*}K_{\hat{h},\hat{v}}+B^{*}F_{\hat{h},\hat{v}}^{*}\text{)}$, ${\text{Ψ}}_{9}^{2}={\left(\bar{BB}\varpi_{2}\right)}^{T}+\varepsilon A_{\hat{h},\hat{v}}^{*}K_{\hat{h},\hat{v}}-P_{\hat{h},\hat{v}}+K_{\hat{h},\hat{v}}+\varepsilon B^{*}F_{\hat{h},\hat{v}}^{*}$, ${\text{Ψ}}_{9}^{3}=-\varepsilon \text{He(}P_{\hat{h},\hat{v}}-\bar{BB}\varpi_{2}\text{)}$, ${\text{Ψ}}_{9}^{4}={\left(\bar{BB}\varpi_{1}\right)}^{T}+A_{h,v}^{*}K_{\hat{h},\hat{v}}-A_{\hat{h},\hat{v}}^{*}K_{\hat{h},\hat{v}}$, ${\text{Ψ}}_{9}^{5}=\varepsilon {\left(\bar{BB}\varpi_{1}\right)}^{T}-L_{\hat{h},\hat{v}}^{*}C_{\hat{h}}^{*}T_{\hat{h}}^{*}R_{2\hat{h},\hat{v}}$, ${\text{Ψ}}_{9}^{6}=K_{\hat{h},\hat{v}}+\varepsilon_{1}{\left(\bar{BB}\varpi_{1}\right)}^{T}$, ${\text{Ψ}}_{9}^{7}=\varepsilon_{1}\varepsilon {\left(\bar{BB}\varpi_{1}\right)}^{T}$, ${\text{Ψ}}_{9}^{8}=-K_{\hat{h},\hat{v}}+\varepsilon_{2}{\left(\bar{BB}\varpi_{2}\right)}^{T}$, ${\text{Ψ}}_{9}^{9}=\varepsilon_{2}\varepsilon {\left(\bar{BB}\varpi_{2}\right)}^{T}-T_{\hat{h}}^{*}R_{2,\hat{h},\hat{v}}$, $\varpi_{1}=L_{\hat{h},\hat{v}}^{*}C_{h}^{*}T_{h}^{*}R_{1,\hat{h},\hat{v}}$, $\varpi_{2}=L_{\hat{h},\hat{v}}^{*}C_{\hat{h}}^{*}T_{\hat{h}}^{*}R_{2,\hat{h},\hat{v}}$, $\varpi_{3}=\text{Δ}L_{\hat{h},\hat{v}}^{*}C_{h}^{*}T_{h}^{*}R_{1,\hat{h},\hat{v}}$, and $\varpi_{4}=\text{Δ}L_{\hat{h},\hat{v}}^{*}C_{\hat{h}}^{*}T_{\hat{h}}^{*}R_{2,\hat{h},\hat{v}}$.

Because $\text{Δ}L_{\hat{h},\hat{v}}^{*}=M_{L}^{*}S_{L}N_{L}^{*}L_{\hat{h},\hat{v}}^{*}$, $\text{Δ}F_{\hat{h},\hat{v}}^{*}=M_{F}S_{F}N_{F}F_{\hat{h},\hat{v}}^{*}$, and ΔA^*^ = *MF*(*t*)*N*, inequality (40) can be rewritten as:


(41) $$\begin{array}{l} {\text{Ψ}}_{9}+\text{He}\left({\text{M}}_{1}{\text{S}}_{\text{F}}{\text{N}}_{1}\text{+}{\text{M}}_{2}\text{F}\left(\text{t}\right){\text{N}}_{2}\text{+}{\text{M}}_{3}{\text{S}}_{\text{L}}{\text{N}}_{3}\right)<\;0, \end{array}$$

$$\begin{array}{c} M_{1}={\left[\begin{array}{ccccc} B^{*}M_{F} & \varepsilon B^{*}M_{F} & 0 & 0 & 0 \end{array}\right]}^{T},N_{1}=\left[\begin{array}{ccccc} N_{F}F_{\hat{h},\hat{v}}^{*} & 0 & 0 & 0 & 0 \end{array}\right],\;M_{2}={\left[\begin{array}{ccccc} 0 & 0 & M & 0 & 0 \end{array}\right]}^{T},\\ N_{2}=\left[\begin{array}{ccccc} NK_{\hat{h},\hat{v}} & 0 & NQ_{\hat{h},\hat{v}} & 0 & 0 \end{array}\right],M_{3}={\left[\begin{array}{ccccc} \bar{BB}M_{L}^{*} & \varepsilon \bar{BB}M_{L}^{*} & -M_{L}^{*} & 0 & 0 \end{array}\right]}^{T},\\ N_{3}=\left[\begin{array}{ccccc} 0 & N_{L}^{*}L_{\hat{h},\hat{v}}^{*}C_{\hat{h}}^{*}T_{\hat{h}}^{*}R_{2,\hat{h},\hat{v}} & N_{L}^{*}L_{\hat{h},\hat{v}}^{*}C_{h}^{*}T_{h}^{*}R_{1,\hat{h},\hat{v}} & \varepsilon_{1}N_{L}^{*}L_{\hat{h},\hat{v}}^{*}C_{h}^{*}T_{h}^{*}R_{1,\hat{h},\hat{v}} & \varepsilon_{2}N_{L}^{*}L_{\hat{h},\hat{v}}^{*}C^{*}T^{*}R_{1,\hat{h},\hat{v}} \end{array}\right]. \end{array}$$

Based on lemma 2, (41) holds if there are positive real scalars $\varepsilon_{3,\hat{h},\hat{v}}$, $\varepsilon_{4,\hat{h},\hat{v}}^{A}$, $\varepsilon_{4,\hat{h},\hat{v}}^{E}$, and $\varepsilon_{\text{5,}\hat{h},\hat{v}}$that satisfy the following relation:


(42) $$\begin{array}{l} {\text{Ψ}}_{9}+M_{1}\varepsilon_{3,\hat{h},\hat{v}}M_{1}^{T}+N_{1}^{T}\varepsilon_{3,\hat{h},\hat{v}}^{-1}N_{1}+M_{2}\varepsilon_{4,\hat{h},\hat{v}}M_{2}^{T}+N_{2}^{T}\varepsilon_{4,\hat{h},\hat{v}}^{-1}N_{2}+M_{3}\varepsilon_{5,\hat{h},\hat{v}}M_{3}^{T}+N_{3}^{T}\varepsilon_{5,\hat{h},\hat{v}}^{-1}N_{3}<0, \end{array}$$

where $\varepsilon_{4,\hat{h},\hat{v}}=\left[\begin{array}{cc} \varepsilon_{4,\hat{h},\hat{v}}^{A}I & 0\\ 0 & \varepsilon_{4,\hat{h},\hat{v}}^{E}I \end{array}\right]$. Using the Schur complement in lemma 1, (42) holds if and only if the following relation set is satisfied:


(43) $$\begin{array}{l} \left[\begin{array}{cccccccc} {\text{Ψ}}_{10}^{1} & * & * & * & * & * & * & *\\ {\text{Ψ}}_{10}^{2} & {\text{Ψ}}_{10}^{3} & * & * & * & * & * & *\\ {\text{Ψ}}_{10}^{4} & {\text{Ψ}}_{10}^{5} & {\text{Ψ}}_{10}^{6} & * & * & * & * & *\\ {\text{Ψ}}_{10}^{7} & \varepsilon_{1}\varepsilon {\left(\bar{BB}\varpi_{1}\right)}^{T} & {\text{Ψ}}_{10}^{8} & {\text{Ψ}}_{10}^{9} & * & * & * & *\\ {\text{Ψ}}_{10}^{10} & {\text{Ψ}}_{10}^{11} & -\varepsilon_{2}\varpi_{2}^{T} & 0 & -\varepsilon_{2}\text{He}{\left(T_{\hat{h}}^{*}R_{2,\hat{h},\hat{v}}\right)}^{T} & * & * & *\\ N_{F}F_{\hat{h},\hat{v}}^{*} & 0 & 0 & 0 & 0 & -\varepsilon_{3,\hat{h},\hat{v}}I & * & *\\ NK_{\hat{h},\hat{v}}^{*} & 0 & NQ_{\hat{h},\hat{v}} & 0 & 0 & 0 & -\varepsilon_{4,\hat{h},\hat{v}}I & *\\ 0 & {\text{Ψ}}_{10}^{12} & {\text{Ψ}}_{10}^{13} & {\text{Ψ}}_{10}^{14} & \varepsilon_{2}N_{L}^{*}L_{\hat{h},\hat{v}}^{*}C^{*}T^{*}R_{1,\hat{h},\hat{v}} & 0 & 0 & -\varepsilon_{5,\hat{h},\hat{v}}I \end{array}\right]<0 \end{array}$$

where ${\text{Ψ}}_{10}^{1}=\text{He(}A_{\hat{h},\hat{v}}^{*}K_{\hat{h},\hat{v}}+B^{*}F_{\hat{h},\hat{v}}^{*}\text{)}+B^{*}M_{F}\varepsilon_{3,\hat{h},\hat{v}}M_{F}^{T}B^{*T}+\bar{BB}M_{L}^{*}\varepsilon_{5,\hat{h},\hat{v}}M_{L}^{*T}{\bar{BB}}^{T}$, ${\text{Ψ}}_{10}^{2}={\left(\bar{BB}\varpi_{2}\right)}^{T}+K_{\hat{h},\hat{v}}$$+\varepsilon A_{\hat{h},\hat{v}}^{*}K_{\hat{h},\hat{v}}-P_{\hat{h},\hat{v}}+\varepsilon B^{*}F_{\hat{h},\hat{v}}^{*}+\varepsilon B^{*}M_{F}{\;\varepsilon \;}_{3,\hat{h},\hat{v}}M_{F}^{T}B^{*T}+\varepsilon \bar{BB}M_{L}^{*}{\;\varepsilon \;}_{5,\hat{h},\hat{v}}M_{L}^{*T}{\bar{BB}}^{T}$, ${\text{Ψ}}_{10}^{3}=\varepsilon^{2}B^{*}M_{F}{\;\varepsilon \;}_{3,\hat{h},\hat{v}}M_{F}^{T}B^{*T}$$+\varepsilon^{2}\bar{BB}M_{L}^{*}{\;\varepsilon \;}_{5,\hat{h},\hat{v}}M_{L}^{*T}{\bar{BB}}^{T}-\varepsilon \text{He}\left(P_{\hat{h},\hat{v}}^{T}-{\left(\bar{BB}\varpi_{2}\right)}^{T}\right)$, ${\text{Ψ}}_{10}^{4}={\left(\bar{BB}\varpi_{1}\right)}^{T}-M_{L}^{*}{\;\varepsilon \;}_{5,\hat{h},\hat{v}}M_{L}^{*T}{\bar{BB}}^{T}+A_{h,v}^{*}K_{\hat{h},\hat{v}}-$$A_{\hat{h},\hat{v}}^{*}K_{\hat{h},\hat{v}}$, ${\text{Ψ}}_{10}^{5}=\varepsilon {\left(\bar{BB}\varpi_{1}\right)}^{T}-\varepsilon M_{L}^{*}{\;\varepsilon \;}_{5,\hat{h},\hat{v}}M_{L}^{*T}{\bar{BB}}^{T}-L_{\hat{h},\hat{v}}^{*}C_{\hat{h}}^{*}T_{\hat{h}}^{*}R_{2\hat{h},\hat{v}}$, ${\text{Ψ}}_{10}^{6}=\;\text{He}\left(A_{h,v}^{*}Q_{\hat{h},\hat{v}}-\varpi_{1}\right)+$$M{\;\varepsilon \;}_{4,\hat{h},\hat{v}}M^{T}+M_{L}^{*}{\;\varepsilon \;}_{5,\hat{h},\hat{v}}M_{L}^{*T}$, ${\text{Ψ}}_{10}^{7}=K_{\hat{h},\hat{v}}+\varepsilon_{1}{\left(\bar{BB}\varpi_{1}\right)}^{T}$, ${\text{Ψ}}_{10}^{8}=Q_{\hat{h},\hat{v}}-\varepsilon_{1}\varpi_{1}^{T}-T_{h}^{*}R_{1,\hat{h},\hat{v}}$,${\text{Ψ}}_{10}^{9}=$$-\varepsilon_{1}\text{He}{\left(T_{h}^{*}R_{1,\hat{h},\hat{v}}\right)}^{T}$,${\text{Ψ}}_{10}^{10}=-K_{\hat{h},\hat{v}}+\varepsilon_{2}{\left(\bar{BB}\varpi_{2}\right)}^{T}$, ${\text{Ψ}}_{10}^{11}=\varepsilon_{2}\varepsilon {\left(\bar{BB}\varpi_{2}\right)}^{T}-T_{\hat{h}}^{*}R_{2,\hat{h},\hat{v}}$, ${\text{Ψ}}_{10}^{12}=N_{L}^{*}L_{\hat{h},\hat{v}}^{*}C_{\hat{h}}^{*}T_{\hat{h}}^{*}R_{2,\hat{h},\hat{v}}$,${\text{Ψ}}_{10}^{13}=N_{L}^{*}L_{\hat{h},\hat{v}}^{*}C_{h}^{*}T_{h}^{*}R_{1,\hat{h},\hat{v}}$,${\text{Ψ}}_{10}^{14}=\varepsilon_{1}N_{L}^{*}L_{\hat{h},\hat{v}}^{*}C_{h}^{*}T_{h}^{*}R_{1,\hat{h},\hat{v}}$.

It is assumed that $S^{*}=\left[\begin{array}{cc} S_{1}^{*} & S_{2}^{*} \end{array}\right]$; thus, the matrix $\bar{BB}$ can be formulated as:


(44) $$\begin{array}{l} \bar{BB}=\left[\begin{array}{cc} I & 0\\ -B{\left(S_{2}^{*}B\right)}^{-1}S_{1}^{*} & I-B{\left(S_{2}^{*}B\right)}^{-1}S_{2}^{*} \end{array}\right]. \end{array}$$

Then, the matrices in inequality (43) can be obtained as follows:


$$\begin{array}{c} L_{\hat{h},\hat{v}}^{*}={\left[\begin{array}{cc} 0 & L_{\hat{h},\hat{v}}^{T} \end{array}\right]}^{T},T_{h}^{*}=\left[\begin{array}{cc} T_{h} & 0\\ 0 & I \end{array}\right],T_{\hat{h}}^{*}=\left[\begin{array}{cc} T_{\hat{h}} & 0\\ 0 & I \end{array}\right],T_{h}^{*}R_{1,\hat{h},\hat{v}}=\left[\begin{array}{cc} T_{h}R_{1,\hat{h},\hat{v}}^{1} & T_{h}R_{1,\hat{h},\hat{v}}^{2}\\ R_{1,\hat{h},\hat{v}}^{3} & R_{1,\hat{h},\hat{v}}^{4} \end{array}\right],*\\ L_{\hat{h},\hat{v}}^{*}C_{h}^{*}T_{h}^{*}R_{1,\hat{h},\hat{v}}=\left[\begin{array}{cc} 0 & 0\\ L_{\hat{h},\hat{v}}C_{h}T_{h}R_{1,\hat{h},\hat{v}}^{1} & 0 \end{array}\right]=\left[\begin{array}{cc} 0_{n\times n} & 0_{n\times n}\\ \left[\begin{array}{cc} {\left(L_{\hat{h},\hat{v}}R_{1}\right)}_{n\times p} & 0_{n\times \left(n-p\right)} \end{array}\right] & 0_{n\times n} \end{array}\right],\\ \bar{BB}L_{\hat{h},\hat{v}}^{*}C_{h}^{*}T_{h}^{*}R_{1,\hat{h},\hat{v}}=\left[\begin{array}{cc} 0_{n\times n} & 0_{n\times n}\\ \left[\begin{array}{cc} {\left(\left(I-B{\left(S_{2}^{*}B\right)}^{-1}S_{2}^{*}\right)L_{\hat{h},\hat{v}}R_{1}\right)}_{n\times p} & 0_{n\times \left(n-p\right)} \end{array}\right] & 0_{n\times n} \end{array},\right]\\ T_{\hat{h}}^{*}R_{2,\hat{h},\hat{v}}=\left[\begin{array}{cc} T_{\hat{h}}R_{2,\hat{h},\hat{v}}^{1} & T_{\hat{h}}R_{2,\hat{h},\hat{v}}^{2}\\ R_{2,\hat{h},\hat{v}}^{3} & R_{2,\hat{h},\hat{v}}^{4} \end{array}\right],\\ L_{\hat{h},\hat{v}}^{*}C_{\hat{h}}^{*}T_{\hat{h}}^{*}R_{2,\hat{h},\hat{v}}=\left[\begin{array}{cc} 0 & 0\\ L_{\hat{h},\hat{v}}C_{\hat{h}}T_{\hat{h}}R_{2,\hat{h},\hat{v}}^{1} & 0 \end{array}\right]=\left[\begin{array}{cc} 0_{n\times n} & 0_{n\times n}\\ \left[\begin{array}{cc} {\left(L_{\hat{h},\hat{v}}R_{1}\right)}_{n\times p} & 0_{n\times \left(n-p\right)} \end{array}\right] & 0_{n\times n} \end{array}\right],\\ \bar{BB}L_{\hat{h},\hat{v}}^{*}C_{h}^{*}T_{h}^{*}R_{2,\hat{h},\hat{v}}=\left[\begin{array}{cc} 0_{n\times n} & 0_{n\times n}\\ \left[\begin{array}{cc} {\left(\left(I-B{\left(S_{2}^{*}B\right)}^{-1}S_{2}^{*}\right)L_{\hat{h},\hat{v}}R_{1}\right)}_{n\times p} & 0_{n\times \left(n-p\right)} \end{array}\right] & 0_{n\times n} \end{array}\right]. \end{array}$$

Therefore, inequality (27) is easily obtained from the above equalities and inequality (43), which completes the proof.

In some special cases, T-S fuzzy descriptor systems may not contain uncertainties, so the following theorem gives sufficient LMI conditions for the stability of the closed-loop system in this case.

Theorem 3: Suppose that the T-S fuzzy descriptor system matrices in (2) satisfy ΔA^*^ = 0 and $C_{h}=C_{\hat{h}}=C^{*}$. Given the constants ε > 0 and ε_1_ > 0, the closed-loop systems are asymptotically stable if positive definite matrices *P*_1_ and *Q*_1_, scalars ε and ε_1_, and a set of matrices *P*_3,*i,k*_, *P*_4,*i,k*_, *F*_*i,k*_, *K*_1,*k*_, *K*_2,*k*_, *K*_3,*i*_, *K*_4,*i*_, *L*_*i,k*_, *R*_1_, $R_{1,i,k}^{11}$, $R_{1,i,k}^{12}$, $R_{1,i,k}^{2}$, $R_{1,i,k}^{3}$, and $R_{1,i,k}^{4}$exist, where *k* ∈ {1, ⋯ , *r*_*e*_} and *i* ∈ {1, ⋯ , *r*}, such that the following LMIs hold:


(45) $$\begin{array}{l} \left[\begin{array}{cccccccc} \text{He(}K_{3,\hat{h}}\text{)} & * & * & * & * & * & * & *\\ {\text{Λ}}_{2,1} & {\text{Λ}}_{2,2} & * & * & * & * & * & *\\ {\text{Λ}}_{3,1} & {\text{Λ}}_{3,2} & -\varepsilon \text{He(}P_{1}\text{)} & * & * & * & * & *\\ {\text{Λ}}_{4,1} & {\text{Λ}}_{4,2} & -\varepsilon P_{3,\hat{h},\hat{v}} & -\varepsilon \text{He(}P_{4,\hat{h},\hat{v}}\text{)} & * & * & * & *\\ 0 & {\text{Λ}}_{5,2} & 0 & \varepsilon {\left(\bar{B}Y_{\hat{h},\hat{v}}\right)}^{T} & \text{He(}Q_{3,\hat{h}}\text{)} & * & * & *\\ {\text{Λ}}_{6,1} & {\text{Λ}}_{6,2} & 0 & 0 & {\text{Λ}}_{6,5} & {\text{Λ}}_{6,6} & * & *\\ 0 & {\text{Λ}}_{7,2} & 0 & \varepsilon_{1}\varepsilon {\left(\bar{B}Y_{\hat{h},\hat{v}}\right)}^{T} & {\text{Λ}}_{7,5} & {\text{Λ}}_{7,6} & {\text{Λ}}_{7,7} & *\\ 0 & 0 & 0 & 0 & {\text{Λ}}_{8,5} & {\text{Λ}}_{8,6} & {\text{Λ}}_{8,7} & {\text{Λ}}_{8,8} \end{array}\right]<0, \end{array}$$

where ${\text{ Λ }}_{2,1}=A_{\hat{h}}K_{1,\hat{v}}-E_{\hat{v}}K_{3,\hat{h}}+BF_{\hat{h},\hat{v}}+K_{4,\hat{h}}^{T}$, ${\text{ Λ }}_{2,2}=\text{He(}A_{\hat{h}}K_{2,\hat{v}}-E_{\hat{v}}K_{4,\hat{h}}\text{)}$, ${\text{ Λ }}_{3,1}=K_{1,\hat{v}}-P_{1}+\varepsilon K_{3,\hat{h}}$, ${\text{ Λ }}_{3,2}=K_{2,\hat{v}}+\varepsilon K_{4,\hat{h}}$, ${\text{ Λ }}_{4,1}=K_{3,\hat{h}}+\varepsilon \left(A_{\hat{h}}K_{1,\hat{v}}-E_{\hat{v}}K_{3,\hat{h}}+BF_{\hat{h},\hat{v}}\right)-P_{3,\hat{h},\hat{v}}$, ${\text{ Λ }}_{4,2}=\varepsilon \left(A_{\hat{h}}K_{2,\hat{v}}-E_{\hat{v}}K_{4,\hat{h}}\right)+K_{4\hat{h}}$$-P_{4,\hat{h},\hat{v}}$, ${\text{ Λ }}_{5,2}={\left(\bar{B}Y_{\hat{h},\hat{v}}\right)}^{T}$, ${\text{ Λ }}_{6,1}=\left(A_{h}-A_{\hat{h}}\right)K_{1,\hat{v}}-\left(E_{v}-E_{\hat{v}}\right)K_{3,\hat{h}}$, ${\text{ Λ }}_{6,2}=\left(A_{h}-A_{\hat{h}}\right)K_{2,\hat{v}}-\left(E_{v}-E_{\hat{v}}\right)K_{4,\hat{h}}$, ${\text{ Λ }}_{6,5}=A_{h}Q_{1}-E_{v}Q_{3,\hat{h}}+Q_{4,\hat{h}}^{T}-Y_{\hat{h},\hat{v}}$, Λ_6,6_ = −He(*E*_*v*_*Q*_4,$\hat{h}$_), ${\text{ Λ }}_{7,2}=\varepsilon_{1}{\left(\bar{B}Y_{\hat{h},\hat{v}}\right)}^{T}$, ${\text{ Λ }}_{7,5}=Q_{1}-TR_{1,\hat{h},\hat{v}}^{1}$, ${\text{ Λ }}_{7,6}=-\varepsilon_{1}{\left(Y_{\hat{h},\hat{v}}\right)}^{T}-TR_{1,\hat{h},\hat{v}}^{2}$, ${\text{ Λ }}_{7,7}=-\varepsilon_{1}\text{He(}TR_{1,\hat{h},\hat{v}}^{1}\text{)}$, ${\text{ Λ }}_{8,5}=Q_{3,\hat{h}}-R_{1,\hat{h},\hat{v}}^{3}$, ${\text{ Λ }}_{8,6}=Q_{4,\hat{h}}-R_{1,\hat{h},\hat{v}}^{4}$, ${\text{ Λ }}_{8,7}=-\varepsilon_{1}{\left(TR_{1,\hat{h},\hat{v}}^{2}\right)}^{T}-\varepsilon_{1}R_{1,\hat{h},\hat{v}}^{3}$, ${\text{ Λ }}_{8,8}=-\varepsilon_{1}\text{He(}R_{1,\hat{h},\hat{v}}^{4}\text{)}$, $\bar{B}=I-B{\left(S_{2}^{*}B\right)}^{-1}S_{2}^{*}$, $R_{1,\hat{h},\hat{v}}^{1}=\left[\begin{array}{cc} R_{1} & 0_{p\times \left(n-p\right)}\\ R_{1,\hat{h},\hat{v}}^{11} & R_{1,\hat{h},\hat{v}}^{12} \end{array}\right]$,$R_{1,\hat{h},\hat{v}}^{2}=\left[\begin{array}{c} 0_{p\times n}\\ R_{1,\hat{h},\hat{v}}^{21} \end{array}\right]$, and $Y_{\hat{h},\hat{v}}=\left[\begin{array}{cc} {\left(L_{\hat{h},\hat{v}}R_{1}\right)}_{n\times p} & 0_{n\times \left(n-p\right)} \end{array}\right]$.

Theorem 4: Suppose that the T-S fuzzy descriptor system matrices in (2) satisfy ΔA^*^ = 0 and *C*_*h*_ ≠ *C*_$\hat{h}$_. Given constants ε > 0, ε_1_ > 0 and ε_2_ > 0, the closed-loop systems are asymptotically stable if positive definite matrices *P*_1_ and *Q*_1_ and a set of matrices *P*_3,*i,k*_, *P*_4,*i,k*_, *F*_*i,k*_, *K*_1,*k*_, *K*_2,*k*_, *K*_3,*i*_, *K*_4,*i*_, *L*_*i,k*_, *R*_1_, $R_{1,i,k}^{11}$, $R_{1,i,k}^{12}$, $R_{2,i,k}^{11}$, $R_{2,i,k}^{12}$,$R_{1,i,k}^{2}$, $R_{1,i,k}^{3}$, $R_{1,i,k}^{4}$, $R_{2,i,k}^{2}$, $R_{2,i,k}^{3}$, and $R_{2,i,k}^{4}$ exist, where *i* ∈ {1, ⋯ , *r*} and *k* ∈ {1, ⋯ , *r*_*e*_}, such that the following LMIs hold:


(46) $$\begin{array}{l} \left[\begin{array}{cccccccccc} \text{He(}K_{3,\hat{h}}\text{)} & * & * & * & * & * & * & * & * & *\\ {\text{Λ}}_{2,1} & {\text{Λ}}_{2,2} & * & * & * & * & * & * & * & *\\ {\text{Λ}}_{3,1} & {\text{Λ}}_{3,2} & -\varepsilon \text{He(}P_{1}\text{)} & * & * & * & * & * & * & *\\ {\text{Λ}}_{4,1} & {\text{Λ}}_{4,2} & {\text{Λ}}_{4,3} & {\text{Λ}}_{4,4} & * & * & * & * & * & *\\ 0 & {\text{Λ}}_{5,2} & 0 & {\text{Λ}}_{5,4} & {\text{Λ}}_{5,5} & * & * & * & * & *\\ {\text{Λ}}_{6,1} & {\text{Λ}}_{6,2} & -Y_{\hat{h},\hat{v}} & 0 & {\text{Λ}}_{6,5} & {\text{Λ}}_{6,6} & * & * & * & *\\ K_{1,\hat{v}} & {\text{Λ}}_{7,2} & 0 & {\text{Λ}}_{7,4} & {\text{Λ}}_{7,5} & {\text{Λ}}_{7,6} & {\text{Λ}}_{7,7} & * & * & *\\ K_{3,\hat{h}} & K_{4,\hat{h}} & 0 & 0 & {\text{Λ}}_{8,5} & {\text{Λ}}_{8,6} & {\text{Λ}}_{8,7} & {\text{Λ}}_{8,8} & * & *\\ K_{1,\hat{v}} & {\text{Λ}}_{9,2} & {\text{Λ}}_{9,3} & {\text{Λ}}_{9,4} & 0 & {\text{Λ}}_{9,6} & 0 & 0 & {\text{Λ}}_{9,9} & *\\ K_{3,\hat{h}} & K_{4,\hat{h}} & -R_{2,\hat{h},\hat{v}}^{3} & {\text{Λ}}_{10,4} & 0 & 0 & 0 & 0 & {\text{Λ}}_{10,9} & {\text{Λ}}_{10,10} \end{array}\right]<0, \end{array}$$

where ${\text{ Λ }}_{2,1}=A_{\hat{h}}K_{1,\hat{v}}-E_{\hat{v}}K_{3,\hat{h}}+BF_{\hat{h},\hat{v}}+K_{4,\hat{h}}^{T}$, ${\text{ Λ }}_{2,2}=\text{He(}A_{\hat{h}}K_{2,\hat{v}}-E_{\hat{v}}K_{4,\hat{h}}\text{)}$, ${\text{ Λ }}_{3,1}=K_{1,\hat{v}}+\varepsilon K_{3,\hat{h}}-P_{1}$, ${\text{ Λ }}_{3,2}=K_{2,\hat{v}}+\varepsilon K_{4,\hat{h}}+{\left(\bar{B}Y_{\hat{h},\hat{v}}\right)}^{T}$, ${\text{Λ}}_{4,1}=K_{3,\hat{h}}+\varepsilon \left(A_{\hat{h}}K_{1,\hat{v}}-E_{\hat{v}}K_{3,\hat{h}}+BF_{\hat{h},\hat{v}}\right)-P_{3,\hat{h},\hat{v}}$, ${\text{ Λ }}_{4,2}=K_{4,\hat{h}}-P_{4,\hat{h},\hat{v}}+$$\varepsilon A_{\hat{h}}K_{2,\hat{v}}-\varepsilon E_{\hat{v}}K_{4,\hat{h}}$, ${\text{ Λ }}_{4,3}=-\varepsilon P_{3,\hat{h},\hat{v}}+\varepsilon \bar{B}Y_{\hat{h},\hat{v}}$, ${\text{ Λ }}_{4,4}=-\varepsilon \text{He(}P_{4,\hat{h},\hat{v}}\text{)}$, ${\text{ Λ }}_{5,2}={\left(\bar{B}Y_{\hat{h},\hat{v}}\right)}^{T}$, ${\text{ Λ }}_{5,4}=\varepsilon {\left(\bar{B}Y_{\hat{h},\hat{v}}\right)}^{T}$, Λ_5,5_ = He(*Q*_3,$\hat{h}$_), ${\text{ Λ }}_{6,1}=\left(A_{h}-A_{\hat{h}}\right)K_{1,\hat{v}}-\left(E_{v}-E_{\hat{v}}\right)K_{3,\hat{h}}$, ${\text{ Λ }}_{6,2}=\left(A_{h}-A_{\hat{h}}\right)K_{2,\hat{v}}-\left(E_{v}-E_{\hat{v}}\right)K_{4,\hat{h}}$, Λ_6,5_=$A_{h}Q_{1}-E_{v}Q_{3,\hat{h}}+Q_{4,\hat{h}}^{T}-Y_{\hat{h},\hat{v}}$, Λ_6,6_ = −He(*E*_*v*_*Q*_4,$\hat{h}$_), $\;{\text{ Λ }}_{7,2}=K_{2\hat{v}}+\varepsilon_{1}{\left(\bar{B}Y_{\hat{h},\hat{v}}\right)}^{T}$,${\text{ Λ }}_{7,4}=\varepsilon_{1}\varepsilon {\left(\bar{B}Y_{\hat{h},\hat{v}}\right)}^{T}$, ${\text{ Λ }}_{7,5}=Q_{1}-T_{h}R_{1,\hat{h},\hat{v}}^{1}$, ${\text{ Λ }}_{7,6}=-\varepsilon_{1}{\left(Y_{\hat{h},\hat{v}}\right)}^{T}-T_{h}R_{1,\hat{h},\hat{v}}^{2}$, ${\text{ Λ }}_{7,7}=-\varepsilon_{1}\text{He(}T_{h}R_{1,\hat{h},\hat{v}}^{1}\text{)}$, ${\text{Λ}}_{8,5}=Q_{3,\hat{h}}-R_{1,\hat{h},\hat{v}}^{3}$, Λ_8,6_=$Q_{4,\hat{h}}-R_{1,\hat{h},\hat{v}}^{4}$, ${\text{ Λ }}_{8,7}=-\varepsilon_{1}R_{1,\hat{h},\hat{v}}^{3}-\varepsilon_{1}{\left(T_{h}R_{1,\hat{h},\hat{v}}^{2}\right)}^{T}$, ${\text{ Λ }}_{8,8}=-\varepsilon_{1}\text{He(}R_{1,\hat{h},\hat{v}}^{4}\text{)}$, ${\text{ Λ }}_{9,2}=K_{2,\hat{v}}+\varepsilon_{2}{\left(\bar{BB}Y_{\hat{h},\hat{v}}\right)}^{T}$, ${\text{ Λ }}_{9,3}=-T_{\hat{h}}R_{2,\hat{h},\hat{v}}^{1}$, ${\text{ Λ }}_{9,4}=\varepsilon_{2}\varepsilon {\left(\bar{B}Y_{\hat{h},\hat{v}}\right)}^{T}-T_{\hat{h}}R_{2,\hat{h},\hat{v}}^{2}$, ${\text{ Λ }}_{9,6}=-\varepsilon_{2}{\left(Y_{\hat{h},\hat{v}}\right)}^{T}$, ${\text{ Λ }}_{9,9}=-\varepsilon_{2}\text{He(}T_{\hat{h}}R_{2,\hat{h},\hat{v}}^{1}\text{)}$, ${\text{ Λ }}_{10,4}=-R_{2,\hat{h},\hat{v}}^{4}$,${\text{ Λ }}_{10,9}=-\varepsilon_{2}{\left(T_{\hat{h}}R_{2,\hat{h},\hat{v}}^{2}\right)}^{T}-\varepsilon_{2}R_{2,\hat{h},\hat{v}}^{3}$, ${\text{ Λ }}_{10,10}=-\varepsilon_{2}\text{He(}T_{\hat{h}}R_{2,\hat{h},\hat{v}}^{4}\text{)}$, $\bar{B}=I-B{\left(S_{2}^{*}B\right)}^{-1}S_{2}^{*}$, $R_{1,\hat{h},\hat{v}}^{1}=\left[\begin{array}{cc} R_{1} & 0_{p\times \left(n-p\right)}\\ R_{1,\hat{h},\hat{v}}^{11} & R_{1,\hat{h},\hat{v}}^{12} \end{array}\right]$, $R_{2,\hat{h},\hat{v}}^{1}=\left[\begin{array}{cc} R_{1} & 0_{p\times \left(n-p\right)}\\ R_{2,\hat{h},\hat{v}}^{11} & R_{2,\hat{h},\hat{v}}^{12} \end{array}\right]$, $R_{1,\hat{h},\hat{v}}^{2}=\left[\begin{array}{c} 0_{p\times n}\\ R_{1,\hat{h},\hat{v}}^{21} \end{array}\right]$, $R_{2,\hat{h},\hat{v}}^{2}=\left[\begin{array}{c} 0_{p\times n}\\ R_{2,\hat{h},\hat{v}}^{21} \end{array}\right]$, and $Y_{\hat{h},\hat{v}}=\left[\begin{array}{cc} {\left(L_{\hat{h},\hat{v}}R_{1}\right)}_{n\times p} & 0_{n\times \left(n-p\right)} \end{array}\right]$.

The corresponding proof can be obtained according to the proofs given for theorems 1 and 2, because theorems 3 and 4 are special cases of theorems 1 and 2, respectively. Therefore, the specific process is omitted here.

### Non-Fragile Observer-Based Adaptive Integral Sliding Mode Controller Design

In practical applications, it is difficult to accurately obtain the bounds of unknown uncertainties and perturbations in controller and observer gains. Hence, an adaptive integral sliding mode controller is designed for T-S fuzzy descriptor systems with uncertainties and perturbations.

Theorem 5: Suppose that the T-S fuzzy descriptor system matrices in (2) satisfy ΔA^*^ ≠ 0, and $C_{h}=C_{\hat{h}}=C^{*}$. Assume that matrices *F*_*i,k*_, *K*_1,*k*_, *K*_2,*k*_, *K*_3,*i*_, *K*_4,*i*_, and *L*_*i,k*_, where *k* ∈ {1, ⋯ , *r*_*e*_} and *i* ∈ {1, ⋯ , *r*}, satisfy theorem 1 and ζ > 0. System (2) can be driven to the sliding surface (7) and maintain sliding motion based on the following ISMC equation:


(47) $$\begin{array}{c} u\left(t\right)=F_{\hat{h},\hat{v}}^{*}K_{\hat{h},\hat{v}}^{-1}{\hat{X}}^{*}\left(t\right)-{\left(S^{*}B^{*}\right)}^{-1}(\|S^{*}L_{\hat{h},\hat{v}}^{*}\left(y\left(t\right)-C^{*}{\hat{X}}^{*}\left(t\right)\right)\|+\|S^{*}M\|\|N{\hat{X}}^{*}\left(t\right)\|+\\ \|S^{*}M_{L}\|\|N_{L}L_{\hat{h},\hat{v}}^{*}\left(y\left(t\right)-C^{*}{\hat{X}}^{*}\left(t\right)\right)\|+\zeta )\frac{s_{{\hat{X}}^{*}\left(t\right)}}{\left\|s_{{\hat{X}}^{*}\left(t\right)}\right\|}-\|M_{F}\|\|N_{F}F_{\hat{h},\hat{v}}^{*}K_{\hat{h},\hat{v}}^{-1}{\hat{X}}^{*}\left(t\right)\|\frac{s_{{\hat{X}}^{*}\left(t\right)}}{\left\|s_{{\hat{X}}^{*}\left(t\right)}\right\|}. \end{array}$$

Proof: Consider the following Lyapunov function candidate:


(48) $$\begin{array}{l} V\left(s_{{\hat{X}}^{*}}\left(t\right)\right)=\frac{1}{2}s_{{\hat{X}}^{*}}^{T}\left(t\right)s_{{\hat{X}}^{*}}\left(t\right). \end{array}$$

The derivative of the Lyapunov function candidate (48) can be obtained as:


(49) $$\begin{array}{c} \overset{∙}{V}\left(s_{{\hat{X}}^{*}}\left(t\right)\right)=s_{{\hat{X}}^{*}}^{T}\left(t\right){\dot{s}}_{{\hat{X}}^{*}}\left(t\right)\\ =s_{{\hat{X}}^{*}}^{T}\left(t\right)\left\{S^{*}\left(B^{*}u\left(t\right)+\left(L_{\hat{h},\hat{v}}^{*}+\text{Δ}L_{\hat{h},\hat{v}}^{*}\right)\left(y\left(t\right)-C^{*}{\hat{X}}^{*}\left(t\right)\right)\right)-S^{*}B^{*}\left(F_{\hat{h},\hat{v}}^{*}+\text{Δ}F_{\hat{h},\hat{v}}^{*}\right)K_{\hat{h},\hat{v}}^{-1}{\hat{X}}^{*}\left(t\right)\right\}. \end{array}$$

By substituting (47) to (49), Equation (49) can be rewritten as


(50) $$\begin{array}{c} \overset{∙}{V}\left(s_{{\hat{X}}^{*}}\left(t\right)\right)=s_{{\hat{X}}^{*}}^{T}\left(t\right)\{S^{*}\left(\left(L_{\hat{h},\hat{v}}^{*}+\text{Δ}L_{\hat{h},\hat{v}}^{*}\right)\left(y\left(t\right)-C^{*}{\hat{X}}^{*}\left(t\right)\right)\right)-S^{*}B^{*}\text{Δ}F_{\hat{h},\hat{v}}^{*}K_{\hat{h},\hat{v}}^{-1}{\hat{X}}^{*}\left(t\right)+\\ S^{*}B^{*}\|M_{F}\|\|N_{F}F_{\hat{h},\hat{v}}K_{\hat{h},\hat{v}}^{-1}{\hat{X}}^{*}\left(t\right)\|\frac{s_{{\hat{X}}^{*}}\left(t\right)}{\left\|s_{{\hat{X}}^{*}}\left(t\right)\right\|}-\|S^{*}L_{\hat{h},\hat{v}}^{*}\left(y\left(t\right)-C^{*}{\hat{X}}^{*}\left(t\right)\right)\|\frac{s_{{\hat{X}}^{*}}\left(t\right)}{\left\|s_{{\hat{X}}^{*}}\left(t\right)\right\|}-\\ \|S^{\text{*}}M\|\|N{\hat{X}}^{*}\left(t\right)\|\frac{s_{{\hat{X}}^{*}\left(t\right)}}{\left\|s_{{\hat{X}}^{*}\left(t\right)}\right\|}-\|S^{\text{*}}M_{L}\|\|N_{L}L_{\hat{h},\hat{v}}^{*}\left(y\left(t\right)-C^{*}{\hat{X}}^{*}\left(t\right)\right)\|\frac{s_{{\hat{X}}^{*}\left(t\right)}}{\left\|s_{{\hat{X}}^{*}\left(t\right)}\right\|}\}. \end{array}$$

Then, from ΔA^*^ = *MF*(*t*)*N*, *F*^*T*^(*t*)*F*(*t*) ≤ *I*, $\text{Δ}L_{\hat{h},\hat{v}}^{*}=M_{L}^{*}S_{L}N_{L}^{*}L_{\hat{h},\hat{v}}^{*}$, $S_{L}^{T}S_{L}\leq I$, $\text{Δ}F_{\hat{h},\hat{v}}^{*}=$$M_{F}S_{F}N_{F}F_{\hat{h},\hat{v}}^{*}$, and $S_{F}^{T}S_{F}\leq I$, it follows that:


(51) $$\begin{array}{l} \overset{∙}{V}\left(s_{\hat{X}}\left(t\right)\right)\leq s_{\hat{X}}^{T}\left(t\right)\left\{-\zeta S^{*}B^{*}\frac{{\left(S^{*}B^{*}\right)}^{-1}s_{{\hat{X}}^{*}}\left(t\right)}{\left\|s_{{\hat{X}}^{*}}\left(t\right)\right\|}\right\}\leq -\zeta \|s_{{\hat{X}}^{*}}\left(t\right)\|. \end{array}$$

From the above analysis, T-S fuzzy descriptor systems can reach the desired sliding mode surface in finite time even in the presence of uncertainties and external disturbances.

In some special cases, T-S fuzzy descriptor systems may not contain uncertainties, so the following theorem gives an observer-designed ISMC strategy in this case.

Theorem 6: Suppose that the T-S fuzzy descriptor system matrices in (2) satisfy ΔA^*^ = 0 and $C_{h}=C_{\hat{h}}=C^{*}$. Additionally, assume that matrices *F*_*i,k*_, *K*_1,*k*_, *K*_2,*k*_, *K*_3,*i*_, *K*_4,*i*_, and *L*_*i,k*_, where *k* ∈ {1, ⋯ , *r*_*e*_} and *i* ∈ {1, ⋯ , *r*}, satisfy theorem 3 and that ζ > 0. System (2) can be driven to the sliding surface and maintain sliding motion based on the following ISMC equation:


(52) $$\begin{array}{l} u\left(t\right)=F_{\hat{h},\hat{v}}^{*}K_{\hat{h},\hat{v}}^{-1}{\hat{X}}^{*}\left(t\right)-{\left(S^{*}B^{*}\right)}^{-1}\left(\|S^{*}L_{\hat{h},\hat{v}}^{*}\left(y\left(t\right)-C^{*}{\hat{X}}^{*}\left(t\right)\right)\|+\zeta \right)\frac{s_{{\hat{X}}^{*}}\left(t\right)}{\left\|s_{{\hat{X}}^{*}}\left(t\right)\right\|}. \end{array}$$

The corresponding proof can be obtained according to the proofs given for theorem 5, because theorem 6 is a special case of theorem 5. Therefore, the specific process is omitted here.

## Examples

In this section, a simple numerical example is simulated to verify the effectiveness and superiority of the proposed method. Consider the following T-S fuzzy descriptor system:


(53) $$\begin{array}{l} \sum_{k=1}^{2}v_{k}\left(\xi \left(t\right)\right)\left(E_{k}+\text{Δ}E\right)\dot{x}\left(t\right)=\sum_{i=1}^{2}h_{i}\left(z\left(t\right)\right)\left(\left(A_{i}+\text{Δ}A\right)x\left(t\right)+Bu\left(t\right)\right), \end{array}$$

where $E_{1}=\left[\begin{array}{cc} 1.1 & -0.1\\ -0.2+b & 1.5 \end{array}\right]$, $E_{2}=\left[\begin{array}{cc} 0.9 & -0.1\\ 0.2 & 0.2 \end{array}\right]$, $A_{1}=\left[\begin{array}{cc} -0.2 & -1\\ -0.1 & -1.9 \end{array}\right]$, $A_{2}=\left[\begin{array}{cc} 1+a & 0.6\\ 1.7 & -0.3 \end{array}\right]$,$B=\left[\begin{array}{c} 0.7\\ 0 \end{array}\right]$, $C=\left[\begin{array}{cc} 1 & 0 \end{array}\right]$, *a* ∈ [−10, 2], *b* ∈ [−0.6, 1],$x\left(t\right)={\left[\begin{array}{cc} x_{1}\left(t\right) & x_{2}\left(t\right) \end{array}\right]}^{T}$,$h_{1}\left(z\left(t\right)\right)=x_{2}^{2}\left(t\right)/4$, *h*_2_(*z*(*t*)) = 1 − *h*_1_(*z*(*t*)), $v_{1}\left(\xi \left(t\right)\right)=1/\left(1+x_{2}^{2}\left(t\right)\right)$, and *v*_2_(ξ(*t*)) = 1 − *v*_1_(ξ(*t*)).

Case 1: Δ*E* = 0andΔ*A* = 0

**Figure 2:**
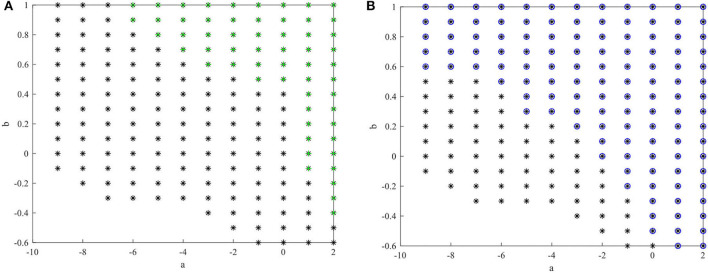
Feasible area for theorem 3 (*) compared with the other methods. **(A)** Feasible area for Theorem 3 (*) and Theorem 2(+) [Ichalal et al., 2011]. **(B)** Feasible area for Theorem 3 (*) and Theorem 1(o) [Asemani and Majd, 2013].

The state feedback controller in (Lin et al., 2006) and the sliding mode controller in (Kchaou et al., 2014) for a class of fuzzy descriptor systems are designed under conditions for which the system states must be measured. Moreover, in (Guerra et al., 2015; Li et al., 2018), the premise variables were dependent on measurable vectors, e.g., the system states *x*_1_(*t*) and*x*_2_(*t*) based on the observer strategy used. However, it is challenging to directly obtain the value of *x*_2_(*t*) with sensors. Therefore, the methods in (Lin et al., 2006; Kchaou et al., 2014; Guerra et al., 2015; Li et al., 2018) cannot be directly applied to T-S fuzzy descriptor systems with unmeasurable system states and premise variables. Several combinations of *a* and *b* are selected to compare the feasible solution region size of theorem 3 with those of theorems 2 (Ichalal et al., 2011) and 1 (Asemani and Majd, 2013), as shown in Figures 2A,B. These Figures show that

the H∞ control method (Asemani and Majd, 2013) is less conservative than the PDC control method based on the traditional quadratic Lyapunov function (Ichalal et al., 2011) for a T-S fuzzy system. The feasible area of the proposed observer-based non-PDC ISMC method for a T-S fuzzy descriptor system is larger than that of the previous two methods. Therefore, in the non-PDC ISMC method, the fuzzy Lyapunov function and descriptor redundancy lead to less conservative results.

When ΔA^*^ = 0, the following values are set: *a* = 1, *b* = 0.5, and ε = 0.0001. Based on theorem 3 and the MATLAB LMI toolbox, the coefficient matrices of the observer-based non-PDC integral sliding mode controller are obtained as follows:


$$\begin{array}{l} L_{11}={\left[\begin{array}{cc} 34.8381 & 4.3355 \end{array}\right]}^{T};L_{12}={\left[\begin{array}{cc} 34.4535 & 1.2693 \end{array}\right]}^{T};\\ L_{21}={\left[\begin{array}{cc} 31.8954 & 2.2550 \end{array}\right]}^{T};L_{22}={\left[\begin{array}{cc} 31.3404 & 0.4542 \end{array}\right]}^{T};\\ F_{11}=\left[\begin{array}{cc} -0.0306 & 0.0082 \end{array}\right];F_{12}=\left[\begin{array}{cc} -0.0200 & 0.0024 \end{array}\right];\\ F_{21}=\left[\begin{array}{cc} -0.0280 & 0.0039 \end{array}\right];F_{22}=\left[\begin{array}{cc} -0.0175 & 0.0051 \end{array}\right]; \end{array}$$

$$\begin{array}{l} K_{11}=\left[\begin{array}{cc} 0.0006 & -0.0007\\ -0.0007 & 0.0022 \end{array}\right];K_{12}=\left[\begin{array}{cc} 0.0006 & -0.0007\\ -0.0007 & 0.0022 \end{array}\right];\\ K_{21}=\left[\begin{array}{cc} -1.3053{\text{e}}^{-6} & -1.9676{\text{e}}^{-7}\\ -1.2372{\text{e}}^{-7} & 9.3139{\text{e}}^{-8} \end{array}\right];\\ K_{22}=\left[\begin{array}{cc} -1.9118{\text{e}}^{-6} & -2.4874{\text{e}}^{-7}\\ 1.4374{\text{e}}^{-8} & 2.4554{\text{e}}^{-7} \end{array}\right];\\ K_{31}=\left[\begin{array}{cc} -0.0031 & 0.0005\\ 0.0008 & -0.0033 \end{array}\right];K_{32}=\left[\begin{array}{cc} -0.0031 & 0.0004\\ 0.0005 & -0.0018 \end{array}\right];\\ K_{41}=\left[\begin{array}{cc} 0.0181 & -0.0007\\ 3.4515{\text{e}}^{-5} & 0.0040 \end{array}\right];K_{42}=\left[\begin{array}{cc} 0.0166 & -0.0006\\ -0.0011 & 0.0021 \end{array}\right]. \end{array}$$

**Figure 3:**
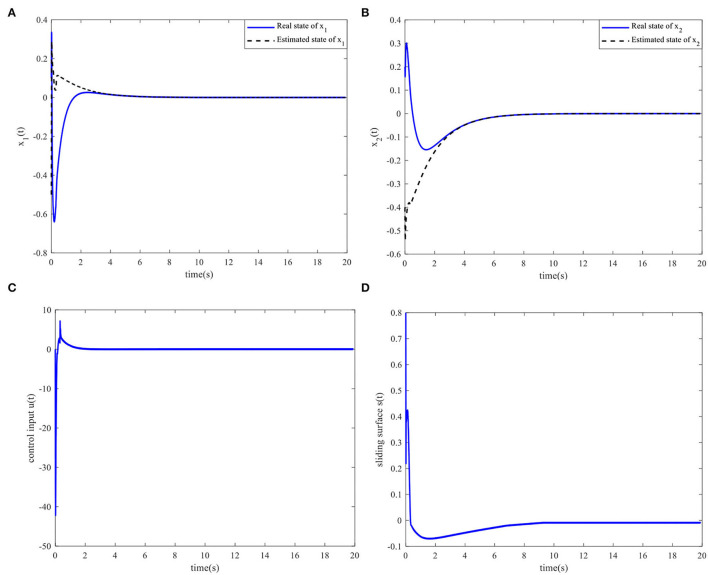
Time responses of the system. **(A)** State *x*_1_(*t*) and the estimated ${\hat{x}}_{1}\left(t\right)$. **(B)** State *x*_2_(*t*) and the estimated ${\hat{x}}_{2}\left(t\right)$. **(C)** Control input. **(D)** Sliding surface.

The controller parameters are selected as ζ = 0.003 and $S^{\text{*}}=\left[\begin{array}{cccc} 1 & 1 & 0.7 & 0 \end{array}\right]$. Assuming the initial states of $x\left(0\right)={\left[\begin{array}{cc} x_{1}\left(0\right) & x_{2}\left(0\right) \end{array}\right]}^{T}={\left[\begin{array}{cc} 0.1 & 0.2 \end{array}\right]}^{T}$and $\hat{x}\left(0\right)={\left[\begin{array}{cc} {\hat{x}}_{1}\left(0\right) & {\hat{x}}_{2}\left(0\right) \end{array}\right]}^{T}={\left[\begin{array}{cc} -0.5 & -0.4 \end{array}\right]}^{T}$, the state responses, control input, and sliding surface for the closed-loop system are shown in Figure 3. The simulation results show that the proposed sliding mode observer can accurately estimate the system state after four s, and that the closed-loop system is asymptotically stable.

Case 2: Δ*E* ≠ 0andΔ*A* ≠ 0.

Assume that the system uncertainties, observer gain perturbation, and controller gain perturbation are expressed by $M_{A}={\left[\begin{array}{cc} 0.06 & 0.02 \end{array}\right]}^{T}$, $M_{E}={\left[\begin{array}{cc} 0.04 & 0.01 \end{array}\right]}^{T}$, $N_{A}=\left[\begin{array}{cc} 0.02 & 0.1 \end{array}\right]$, $N_{E}=\left[\begin{array}{cc} 0.03 & 0.05 \end{array}\right]$, *F*_*A*_(*t*) = 0.5sin(*x*_1_(*t*)), *F*_*E*_(*t*) = 0.2cos(*x*_1_(*t*)), $M_{L}={\left[\begin{array}{cc} 0.05 & 0.2 \end{array}\right]}^{T}$, *N*_*L*_ =$\left[\begin{array}{cc} 0.01 & 0.12 \end{array}\right]$, *M*_*F*_ = 0.1, *N*_*F*_ = 0.2, *S*_*L*_ = 0.5sin(*x*_1_(*t*)), and *S*_*F*_ = 2sin(*x*_1_(*t*))cos(*x*_1_(*t*)). In this case, the parameters are selected as *a* = 1, *b* = 0.5, ε = 0.1, and ε_1_ = 0.001. Moreover, using theorem 1 and the MATLAB LMI toolbox, the following coefficient matrices are obtained:


$$\begin{array}{l} L_{11}={\left[\begin{array}{cc} 42.1615 & 4.6701 \end{array}\right]}^{T};L_{12}={\left[\begin{array}{cc} 41.5437 & 1.4550 \end{array}\right]}^{T};\\ L_{21}={\left[\begin{array}{cc} 39.0960 & 2.5140 \end{array}\right]}^{T};L_{22}={\left[\begin{array}{cc} 37.9514 & 0.5004 \end{array}\right]}^{T};\\ F_{11}=\left[\begin{array}{cc} -0.0349 & 0.0160 \end{array}\right];F_{12}=\left[\begin{array}{cc} -0.0237 & 0.0075 \end{array}\right];\\ F_{21}=\left[\begin{array}{cc} -0.0293 & 0.0070 \end{array}\right];F_{22}=\left[\begin{array}{cc} -0.0126 & 0.0030 \end{array}\right]; \end{array}$$

$$\begin{array}{l} K_{11}=\left[\begin{array}{cc} 0.0018 & -0.0015\\ -0.0015 & 0.0046 \end{array}\right];K_{12}=\left[\begin{array}{cc} 0.0018 & -0.0015\\ -0.0017 & 0.0039 \end{array}\right];\\ K_{21}=\left[\begin{array}{cc} -0.0015 & -0.0004\\ 0.0001 & 0.0002 \end{array}\right];\\ K_{22}=\left[\begin{array}{cc} -0.0022 & -0.0004\\ 0.0012 & 0.0006 \end{array}\right];\\ K_{31}=\left[\begin{array}{cc} -0.0058 & 0.0022\\ 0.0010 & -0.0059 \end{array}\right];K_{32}=\left[\begin{array}{cc} -0.0054 & 0.0015\\ 0.0007 & -0.0031 \end{array}\right];\\ K_{41}=\left[\begin{array}{cc} 0.0227 & -0.0006\\ 0.0006 & 0.0073 \end{array}\right];K_{42}=\left[\begin{array}{cc} 0.0186 & -0.0005\\ -0.0012 & 0.0038 \end{array}\right]. \end{array}$$

It is obvious that different initial system states will lead to different simulation results. Therefore, if the proposed method does not have a wide operating range, the system will not be stable when changing the initial parameters. Therefore, the following initial values of the systems are set to verify the effectiveness of the proposed method for a wide operating range: initial simulation case 1:$x\left(0\right)={\left[\begin{array}{cc} 0.1 & 0.2 \end{array}\right]}^{T}$, $\hat{x}\left(0\right)={\left[\begin{array}{cc} -0.5 & -0.4 \end{array}\right]}^{T}$; initial simulation case 2: $x\left(0\right)={\left[\begin{array}{cc} -0.8 & 0.5 \end{array}\right]}^{T}$, $\hat{x}\left(0\right)={\left[\begin{array}{cc} 0.6 & -0.7 \end{array}\right]}^{T}$; initial simulation case 3: $x\left(0\right)={\left[\begin{array}{cc} 0.8 & -0.5 \end{array}\right]}^{T}$, $\hat{x}\left(0\right)={\left[\begin{array}{cc} -0.6 & 0.7 \end{array}\right]}^{T}$; initial simulation case 4: $x\left(0\right)={\left[\begin{array}{cc} -0.5 & -0.7 \end{array}\right]}^{T}$, $\hat{x}\left(0\right)={\left[\begin{array}{cc} 0.3 & 0.5 \end{array}\right]}^{T}$.

**Figure 4:**
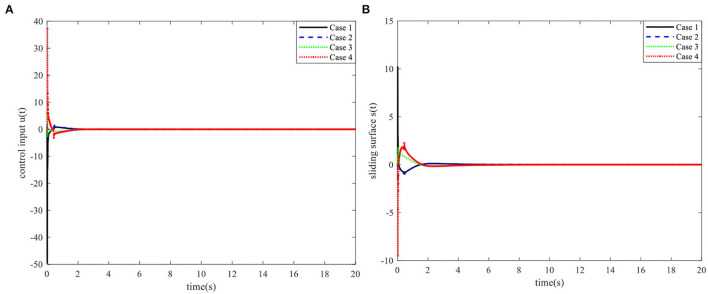
Time responses of the control input and the sliding surface. **(A)** Control input. **(B)** Sliding surface.

**Figure 5:**
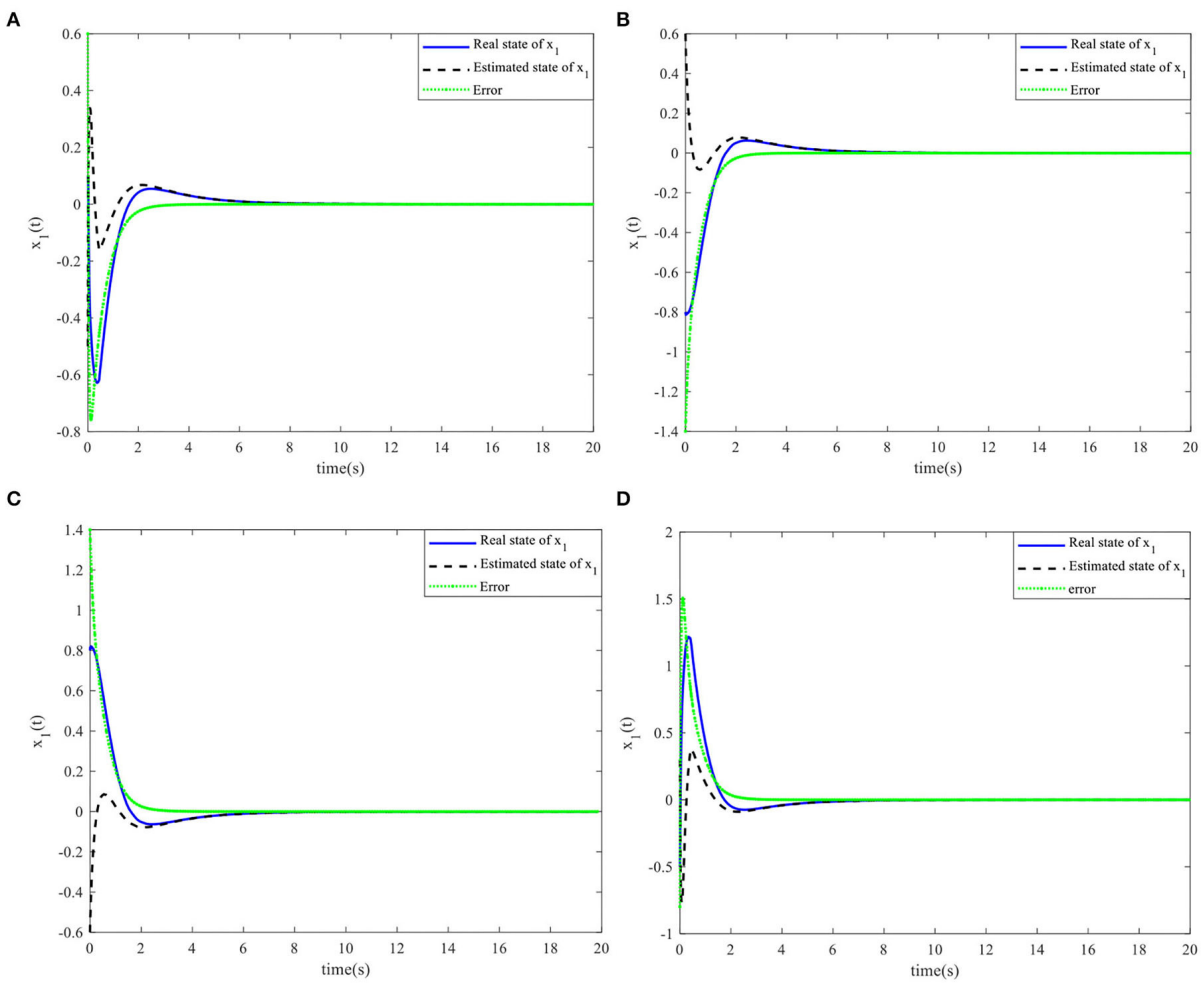
Nonlinear state *x*_1_(*t*) and the estimated ${\hat{x}}_{1}\left(t\right)$. **(A)** Initial simulation case 1. **(B)** Initial simulation case 2. **(C)** Initial simulation case 3. **(D)** Initial simulation case 4.

**Figure 6:**
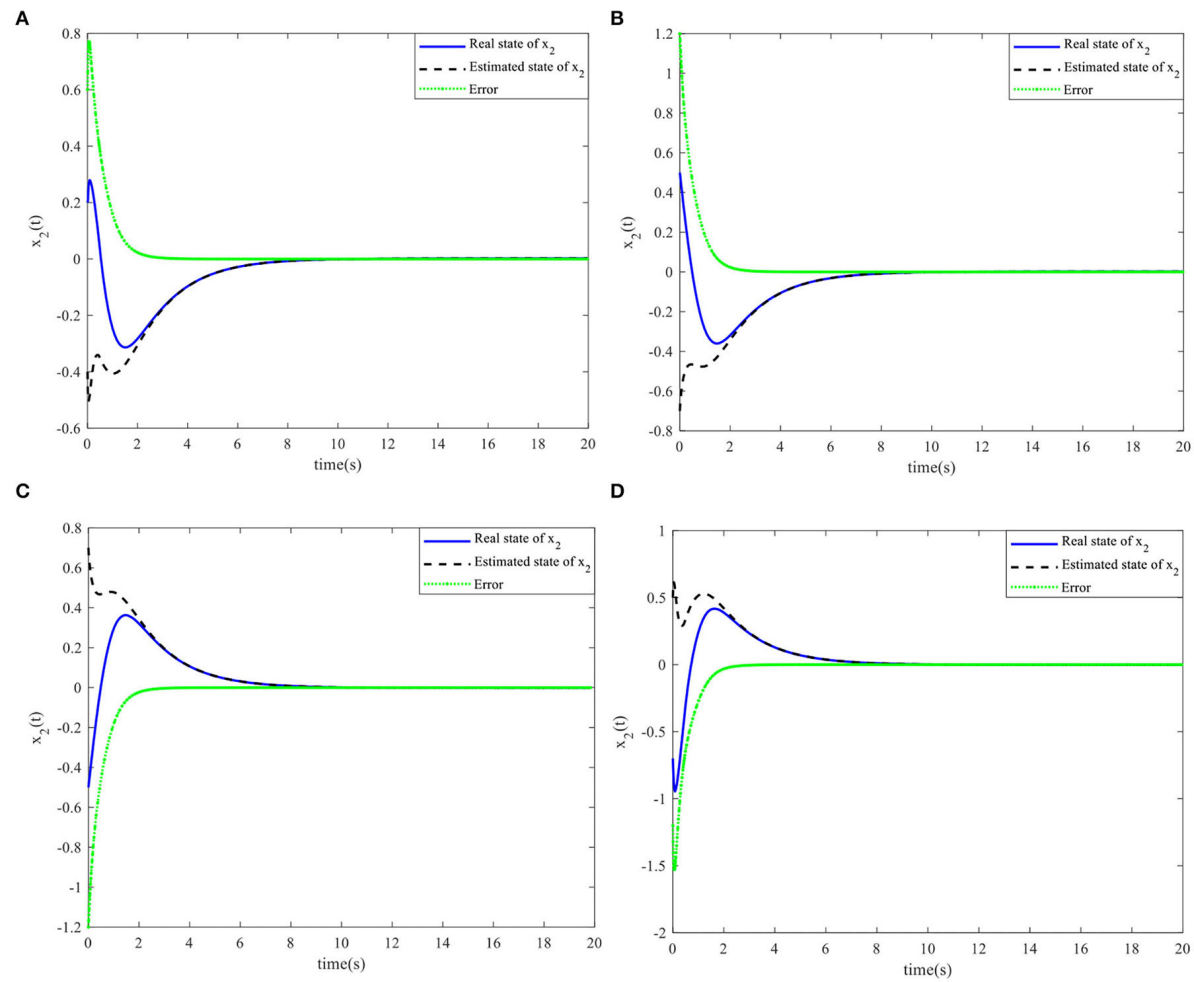
Nonlinear state *x*_2_(*t*) and the estimated ${\hat{x}}_{2}\left(t\right)$. **(A)** Initial simulation case 1. **(B)** Initial simulation case 2. **(C)** Initial simulation case 3. **(D)** Initial simulation case 4.

When the controller parameters are set as ζ = 0.003and $S^{*}=\left[\begin{array}{cccc} 1 & 1 & 0.7 & 0 \end{array}\right]$, the time responses for nonlinear states, the control input, and the sliding mode surface are as shown in Figures 4–6, respectively. The simulation results demonstrate that the system state models display good convergence performance; even when the system has uncertain characteristics, the sliding mode observer can accurately estimate the real states for a nonlinear system with different initial state values, and the designed controller has good robustness and is not fragile to system uncertainties, observer perturbations, and controller perturbations.

## Conclusions

The problem of non-fragile observer-based adaptive ISMC for a class of T-S fuzzy descriptor systems with unmeasurable premise variables is considered in this study. For unmeasurable states, a sliding mode observer is designed, and an integral sliding mode surface is constructed considering the features of the fuzzy sliding mode observer system. Using the Lyapunov theory and designing a fuzzy Lyapunov function, sufficient conditions in terms of LMIs are obtained; additionally, asymptotically stable dynamic estimation error and sliding mode dynamics are achieved. An observer-based ISMC strategy is obtained to meet the reachability conditions. Moreover, a non-fragile observer and a non-fragile adaptive controller are developed such that system uncertainties and perturbations associated with both the observer and the controller can be mitigated. Simulation examples are presented to demonstrate the excellent state estimation performance and effectiveness of the controller.

## Data Availability Statement

The original contributions presented in the study are included in the article/supplementary material, further inquiries can be directed to the corresponding author.

## Author Contributions

XH: conceptualization, writing (original draft), and software. AR: validation and writing (review and editing). YP: investigation and conceptualization. HG: resources and formal analysis. SS: conceptualization, methodology, and supervision.

## Funding

This study was supported in part by the Sichuan Science and Technology Program under grant 2020YFG0211, 2022YFG0094, and 2018GZ0110, the SWJTU Basic Research Cultivation Support Program under grant 2682021ZTPY068, the SWJTU Science and Technology Innovation Project under grant 2682022CX008, the Scientific Research Foundation of the Education Department of Sichuan Province under grant 18CZ0017, the National Key R&D Program of China under grant 2018YFB1201603, and the Innovation Fund of Postgraduate of Xihua University under grants YCJJ2021093 and YCJJ2021088.

## Conflict of Interest

The authors declare that the research was conducted in the absence of any commercial or financial relationships that could be construed as a potential conflict of interest.

## Publisher's Note

All claims expressed in this article are solely those of the authors and do not necessarily represent those of their affiliated organizations, or those of the publisher, the editors and the reviewers. Any product that may be evaluated in this article, or claim that may be made by its manufacturer, is not guaranteed or endorsed by the publisher.
